# Impact of patient and public involvement on enrolment and retention in clinical trials: systematic review and meta-analysis

**DOI:** 10.1136/bmj.k4738

**Published:** 2018-11-28

**Authors:** Joanna C Crocker, Ignacio Ricci-Cabello, Adwoa Parker, Jennifer A Hirst, Alan Chant, Sophie Petit-Zeman, David Evans, Sian Rees

**Affiliations:** 1 Health Experiences Research Group, Nuffield Department of Primary Care Health Sciences, University of Oxford, Oxford OX2 6GG, UK; 2 National Institute for Health Research (NIHR) Oxford Biomedical Research Centre (BRC), John Radcliffe Hospital, Oxford, UK; 3 Balearic Islands Health Research Institute (IdISBa), Palma de Mallorca, Spain; 4 Primary Care Research Unit of Mallorca, Balearic Islands Health Service, Palma de Mallorca, Spain; 5 Ciber de Epidemiologia y Salud Publica (CIBERESP), Madrid, Spain; 6 York Trials Unit, Department of Health Sciences, University of York, York, UK; 7 Nuffield Department of Primary Care Health Sciences, University of Oxford, Oxford, UK; 8 University of the West of England, Bristol, UK; 9 Oxford Academic Health Science Network, Oxford, UK

## Abstract

**Objective:**

To investigate the impact of patient and public involvement (PPI) on rates of enrolment and retention in clinical trials and explore how this varies with the context and nature of PPI.

**Design:**

Systematic review and meta-analysis.

**Data sources:**

Ten electronic databases, including Medline, INVOLVE Evidence Library, and clinical trial registries.

**Eligibility criteria:**

Experimental and observational studies quantitatively evaluating the impact of a PPI intervention, compared with no intervention or non-PPI intervention(s), on participant enrolment and/or retention rates in a clinical trial or trials. PPI interventions could include additional non-PPI components inseparable from the PPI (for example, other stakeholder involvement).

**Data extraction and analysis:**

Two independent reviewers extracted data on enrolment and retention rates, as well as on the context and characteristics of PPI intervention, and assessed risk of bias. Random effects meta-analyses were used to determine the average effect of PPI interventions on enrolment and retention in clinical trials: main analysis including randomised studies only, secondary analysis adding non-randomised studies, and several exploratory subgroup and sensitivity analyses.

**Results:**

26 studies were included in the review; 19 were eligible for enrolment meta-analysis and five for retention meta-analysis. Various PPI interventions were identified with different degrees of involvement, different numbers and types of people involved, and input at different stages of the trial process. On average, PPI interventions modestly but significantly increased the odds of participant enrolment in the main analysis (odds ratio 1.16, 95% confidence interval and prediction interval 1.01 to 1.34). Non-PPI components of interventions may have contributed to this effect. In exploratory subgroup analyses, the involvement of people with lived experience of the condition under study was significantly associated with improved enrolment (odds ratio 3.14 *v* 1.07; P=0.02). The findings for retention were inconclusive owing to the paucity of eligible studies (odds ratio 1.16, 95% confidence interval 0.33 to 4.14), for main analysis).

**Conclusions:**

These findings add weight to the case for PPI in clinical trials by indicating that it is likely to improve enrolment of participants, especially if it includes people with lived experience of the health condition under study. Further research is needed to assess which types of PPI work best in particular contexts, the cost effectiveness of PPI, the impact of PPI at earlier stages of trial design, and the impact of PPI interventions specifically targeting retention.

**Systematic review registration:**

PROSPERO CRD42016043808.

## Introduction

Poor recruitment and retention of patients in trials are major sources of research inefficiency because they delay the delivery of research, inflate its costs, and can lead to biased findings.^1^ ^2^ The top inefficiency in the conduct of trials from recruitment of the first participant to publication of results is failure to meet recruitment targets.^3^ Directors of UK clinical trials units have identified “research into methods to boost recruitment in trials” and “methods to minimise attrition” as the top two priorities for trials methodology research.^4^ In the UK, only 56% of trials funded by the Health Technology Assessment programme recruit their originally specified target sample size, with 32% receiving an extension.^5^ ^6^ Several initiatives aimed at improving recruitment and retention in clinical trials have been established, including the MRC START research programme and Trial Forge.^7^ ^8^ Recruitment and retention interventions identified as meriting formal evaluation include patient and public involvement (PPI).^9^

In the UK, PPI (also known as “public involvement”) has been defined as “research being carried out ‘with’ or ‘by’ members of the public (including patients and carers) rather than ‘to’, ‘about’ or ‘for’ them.”^10^ Trials in the UK have experienced a recent surge in PPI activity, partly because the National Institute for Health Research (NIHR) now expects active PPI in the research it funds.^11^ Patients and members of the public are primarily involved in agenda setting, steering committees, ethical review, protocol development, and piloting.^12^ Many different types of involvement exist, from one person to many people or whole patient organisations, from one-off involvement in a particular aspect of the trial (for example, reviewing draft information for patients or recruiting participants from their communities) to involvement throughout the trial (for example, as members of a trial steering committee), and from involvement with no decision making power (for example, as advisers) to involvement in decision making as equal partners.

Two broad arguments are made for involving patients and members of the public in health research: the moral argument (those affected by, or paying for, research should have a say in what and how it is done) and the consequentialist argument (PPI should improve the quality, efficiency, and impact of research). Because clinical trialists and funders are steeped in a predominantly quantitative, evidence based culture, the consequentialist argument for PPI in clinical trials (for example, that it increases participant enrolment rates) is likely to play an important role in the adoption of meaningful PPI as routine, widespread practice. Hypotheses about how PPI could increase enrolment rates include greater access to potential participants, improved information sheets, more patient centred trial design, more relevant research questions, and peer endorsement of research.^13^ ^14^ ^15^ ^16^ One observational study of 114 trials reported a doubled odds of successful recruitment associated with “consumer input,” but this did not attain statistical significance (odds ratio 2.00, 95% confidence interval 0.36 to 10.05).^17^ A more recent observational study reported a statistical association between PPI and success of recruitment among UK mental health research studies,^13^ but many potential confounding factors could not be controlled for, and information about the nature of PPI in the included studies was lacking. Exploring the effectiveness of PPI practices to improve recruitment and retention of trial participants has been identified as one of the top research priorities for PPI in trials.^18^

This review aimed to measure the impact of PPI interventions on recruitment (specifically participant enrolment) and retention in clinical trials. A secondary objective was to explore how this impact varies according to context (for example, patient population, recruitment setting, trial treatment/intervention) and the nature of the PPI intervention (for example, activities, involvement model, and other PPI characteristics).

## Methods

### Searches

Our systematic literature review followed the PRISMA statement.^19^ We did a systematic electronic search in the following databases (last updated October 2017): Medline, Science Citation Index, Social Science Citation Index, Embase, PsychINFO, Cochrane Library, CINAHL, and Health Expectations journal. We constructed the search strategy by combining keywords within four topic domains: clinical trials, PPI, enrolment or retention of participants, and potential outcomes/change (see appendix 1). In addition to the electronic database search, we searched the INVOLVE Evidence Library for any papers pertaining to the impact of public involvement on health or public health research,^20^ as well as the ClinicalTrials.gov and WHO ICTRP clinical trial registries.

### Screening and study selection

We conceptualised PPI as a complex intervention,^21^ involving human behaviours and often multiple interactive components. We included papers that quantitatively evaluated the impact of a PPI intervention, compared with no intervention or another non-PPI intervention, on enrolment and/or retention rates in a clinical trial or trials in any patient population (see eligibility criteria in table 1 for further details). We defined “PPI intervention” as an intervention that was, or included as an active component, any form of PPI consistent with the INVOLVE definition of public involvement: “research being carried out ‘with’ or ‘by’ members of the public rather than ‘to’, ‘about’ or ‘for’ them,” where the term public includes patients, potential patients, carers, and people who use health and social care services, as well as people from organisations that represent people who use services.^10^ This included interventions not necessarily labelled or conceptualised as “PPI” by the study authors (for example, user testing, peer recruitment, and community based participatory research). We included interventions in which PPI was integrated with additional components inseparable from the PPI (such as involvement of other stakeholders) because this is consistent with the way patients are often involved in practice (for example, being part of an advisory group). Hereafter, we refer to such components as “non-PPI components” of interventions.

**Table 1:** Study eligibility criteria

Parameter	Eligibility criteria
Population	Potential clinical trial participants in any patient population
Intervention	A trial methodology intervention that was, or included as an active component, any of kind PPI consistent with the INVOLVE definition of public involvement: “research being carried out ‘with’ or ‘by’ members of the public rather than ‘to’, ‘about’ or ‘for’ them.”^10^ The term “public” includes patients, potential patients, carers, and people who use health and social care services, as well as people from organisations that represent people who use services. The PPI contributor(s) had to be either a patient, a carer, or a lay member of the public; research or healthcare professionals with the health condition under investigation were included as PPI, but research or healthcare professionals only sharing a characteristic with the target population other than health condition (eg, ethnicity, sex, age) were excluded. Qualitative research was included as a form of patient or public consultation, as this was previously deemed PPI in an INVOLVE report of impact of PPI.^16^ However, as qualitative research is excluded from many definitions of PPI, a sensitivity analysis without this type of study was done
Comparator	No intervention or another trial methodology intervention with no PPI. Studies with no direct comparison group were excluded (eg, those comparing enrolment and/or retention rates against what might be expected for that patient population)
Outcome	Enrolment and/or retention rate, defined as the proportion of potential participants enrolled and the proportion of enrolled participants retained, respectively. Enrolment included giving consent to take part or being randomised to the trial. Studies that assessed hypothetical participation or willingness to participate in clinical trials, rather than actual enrolment in a trial, were excluded. Retention included adherence to a treatment programme and/or follow-up procedures. At the start of data extraction for the meta-analyses, for pragmatic reasons a decision was taken to exclude studies with no appropriate enrolment rate denominator (eg, enrolment reported as absolute numbers rather than rates). This led to the retrospective exclusion of some studies that had been included during initial screening
Context	Clinical trial or trials, defined by the World Health Organization as “any research study that prospectively assigns human participants or groups of humans to one or more health-related interventions to evaluate the effects on health outcomes.” Interventions include but are not restricted to drugs, cells and other biological products, surgical procedures, radiological procedures, devices, behavioural treatments, process of care changes, preventive care, etc. This definition includes phase I to phase IV trials.^22^ For inclusion in the review, the primary outcome had to be a measure of health status; studies of trials with a behavioural or other non-clinical primary outcome were excluded
Study design	Non-randomised (including observational) studies as well as randomised studies were included, as randomisation would not be practical for many PPI interventions

PPI=patient and public involvement.

A review restricted to randomised controlled trials would give an incomplete summary of the impact of PPI, as many types of PPI interventions (for example, patient involvement in the early stages of trial design) are not amenable to randomisation; we therefore included non-randomised as well as randomised evaluations, with a plan for assessing risk of bias. We accepted all non-randomised study designs (provided there was a direct comparison group), including non-randomised controlled trials, controlled and uncontrolled before-after studies, and observational studies. Comparison groups were patients unexposed to the PPI intervention (for example, before its introduction) or patients exposed to an alternative intervention with no PPI (for example, recruitment via healthcare professionals). The evaluation did not have to be the study authors’ primary research question. We put no limits on publication date or language.

Initially, one reviewer (JC) screened all titles and abstracts for potentially eligible papers and subsequently assessed full text papers against the eligibility criteria. Another reviewer (SR) supervised this process and provided advice when there was uncertainty about eligibility. Later, we received funding for a second reviewer (IRC) to independently screen all records in addition to JC. At the end of this process, JC and IRC compared their results in terms of studies included and excluded. Discrepancies were discussed and the opinion of a third reviewer (AP) was sought when necessary to achieve consensus. We contacted authors to provide further information when confirmation of eligibility was needed.

AP and IRC also did forwards and backwards citation searches by hand searching reference lists of included studies and review articles and using the “cited by” function in Scopus. Any potentially eligible papers were double screened for eligibility by JC.

### Data extraction

Using a standardised data extraction form in Microsoft Access, one of three reviewers (JC, AP, or IRC) extracted qualitative information from each paper about the context of the trial, the nature of PPI interventions, and the nature and findings of evaluations. This form was piloted and revised by JC and AP in the early stages. Two reviewers (JC and IRC) then independently extracted quantitative data from included papers on the primary outcomes (enrolment and retention), context, and PPI intervention into a standardised Microsoft Excel spreadsheet for the meta-analyses. For enrolment, we extracted the number of people invited, approached, or reached during the recruitment period (denominator) and the number who consented to take part in the clinical trial (numerator). We included the proxy denominator “total number of participants,” where the intervention targeted a subgroup within the trial population (such as a minority ethnic group or specific geographical region) and the evaluation compared subgroup proportions with and without the intervention. For retention, we extracted the number of people who consented to take part (denominator) and the number who adhered to the trial protocol and/or completed follow-up for the longest period of time investigated by the authors (numerator). We chose the context and intervention variables a priori (table 2) because we considered them to be potentially influential on enrolment and retention outcomes, they are sometimes or often reported in study publications, and, if categorical, they could be split into no more than two or three categories (owing to the small overall sample size). This is consistent with recommendations that systematic reviews of complex interventions include typologies of the structural characteristics of the intervention and, where few or no typologies exist, that face validity for categorisation be provided by experts working in the field.^23^ We considered theories of change underpinning interventions to be potentially important, but we could not categorise them appropriately for inclusion in this analysis. We are doing a realist analysis on the same sample of studies to shed light on the underlying theory and mechanisms of impact of the included interventions (to be published separately).

**Table 2:** Variables extracted and included in subgroup analyses

Variable category	Variable	Format	Description/additional information
Outcomes data	Enrolment rate denominator	Pre-eligibility or post-eligibility screening	An intervention might increase the number of recruits, but not necessarily the number of eligible recruits, if enrolment was measured before screening for eligibility occurred. Where both pre-screening and post-screening enrolment figures were provided by the authors, both were extracted but only the pre-eligibility figure was used in the primary meta-analysis as this spans a greater period of the recruitment process. Subgroup analyses tested whether a difference existed between pre-eligibility and post-eligibility enrolment findings
Contextual data	Trial recruitment setting	Healthcare, community, or mixed (both settings)	“Healthcare” means participants were recruited via contact or association with a healthcare service
	Trial intervention type	Simple, complex, or multiple	“Simple” included drugs, other biological products, and medical devices. “Complex” included surgical procedures and behavioural, psychological, educational, and health service interventions. “Multiple” means that trials of both types of interventions were included in the study
	PPI in choosing research question/topic	Yes or no	PPI in choosing the research question or topic might improve enrolment owing to increased relevance/importance to the target population. If not reported in the paper or accompanying papers, and if study authors did not respond to requests for further information, it was assumed that the answer was “no”
PPI intervention characteristics	Timing/activity	(1) Designing recruitment or retention strategy. (2) Developing patient-facing information. (3) Directly approaching/recruiting or retaining participants	Timing of the start of PPI intervention/first PPI activity. Earlier involvement might lead to greater improvements for enrolment/retention. “Patient-facing information” included paper and online materials and verbal messaging
	No of above activities targeted by PPI intervention (1-3)	1, 2, or 3	More extensive involvement might lead to greater improvements for enrolment/retention
	PPI intervention chosen/designed specifically to increase enrolment or retention	Yes or no	An intervention chosen or designed with this specific purpose may be more effective
	PPI model	One-off, intermittent, or full team membership	“One-off”=time limited, single phase, or single task (eg, a focus group). “Intermittent”=involved periodically during the life of the trial (eg, an ongoing advisory group). “Full team membership”=PPI contributors considered part of the research team (eg a grant co-applicant, co-investigator, research partner, or employed recruiter)
	No of PPI contributors involved	1-2 or ≥3	A group of PPI contributors may provide more diverse perspectives than 1 or 2 individuals, the latter being common practice in UK trial steering committees
	Lived experience of condition under study	Yes or no	At least one PPI contributor had lived experience (as patient or carer) of the health condition being targeted by the trial. If study authors did not indicate that lay/public contributors were patients or had lived experience of the target condition, and did not respond to requests for clarification, it was assumed that the answer was “no”
	PPI visible to potential trial participants	Yes or no	This means that potential trial participants would have known about the PPI, either through direct interaction with PPI contributors or from information about their involvement in the trial

PPI=patient and public involvement.

Discrepancies between the two data extractors (JC and IRC) were discussed, and the opinion of a third reviewer (AP) was sought if necessary to achieve consensus. We sought additional or accompanying papers where necessary to obtain the data we needed (for example, papers describing the contextual clinical trial or the development of the intervention) and contacted authors to provide further information when insufficient data were reported in available papers.

### Risk of bias assessment

Two reviewers (JC and IRC) independently assessed the risk of bias of the studies included in meta-analyses by using the Cochrane Risk of Bias tool for randomised studies and the ROBINS-I tool for non-randomised studies (with pre-specified potential confounding domains of time, funder, and patient population).^24^ ^25^ Discrepancies were discussed and a third reviewer consulted if necessary to achieve consensus. The studies were assessed for risk of bias in relation to our review question, not the study authors’ primary research question (which often differed from ours, particularly for the non-randomised studies).

### Meta-analyses

The only criterion for carrying out meta-analyses was the availability of sufficient outcomes. We took the view that any amount of statistical heterogeneity would be acceptable,^26^ and we considered that, even in the presence of high heterogeneity, an estimate of the average effect of PPI interventions across studies and the statistical significance of this effect were worth reporting. We did two separate meta-analyses to determine the average impact of PPI interventions on enrolment and retention. We combined the numbers of participants enrolled and retained with and without PPI by using a random effects DerSimonian and Laird meta-analysis to report odds ratios. We used the Hartung-Knapp-Sidik-Jonkman variance correction to calculate 95% confidence intervals reflecting the uncertainty in heterogeneity estimates.^27^ ^28^ ^29^ We examined statistical heterogeneity by using the I^2^ statistic and by calculating approximate 95% prediction intervals (which indicate a predicted range for the true effect of a PPI intervention in an individual study)^30^ using methods reported by Higgins et al.^31^ Because of high methodological and statistical heterogeneity across non-randomised studies, we made a post-hoc decision to present findings from randomised studies only as our main analysis. We then did a secondary analysis including non-randomised studies as well as randomised studies. Where multiple non-PPI recruitment strategies had been used within a non-randomised study, we pooled the data for comparison with the PPI recruitment strategy. Where multiple PPI interventions had been compared within a study, we included both interventions as separate comparisons in the meta-analysis and split numbers of participants in the comparator group equally across the two intervention arms.

We did pre-planned subgroup analyses on all included studies (randomised and non-randomised combined) to explore the influence of context and characteristics of the PPI intervention on the association between PPI interventions and enrolment rates and to investigate sources of heterogeneity (table 2). We used univariable meta-regression to determine whether differences between subgroups were statistically significant.

We did sensitivity analyses on both the main analysis (randomised studies only) and the secondary analysis (randomised and non-randomised studies combined). These excluded studies at high risk of bias, studies with small sample sizes (n<100), PPI interventions that included additional non-PPI components, PPI interventions that were formal qualitative research (and therefore not universally classified as PPI), and studies using a proxy denominator to measure enrolment rate (see table 2).

We used Peters’ test to examine small study effects.^32^ ^33^ As only two included studies investigated the cost per participant enrolled of PPI versus non-PPI interventions, we did not do a meta-analysis for this outcome. We used Stata 14.0SE for all analyses, with a threshold of P<0.05 to determine statistical significance.

### Patient and public involvement

The idea for this review emerged from meetings with an advisory panel for JC’s research fellowship in PPI impact assessment, which included two patient partners (including author AC). The patient partners were involved in the group to ensure that the research was relevant to, and informed by the perspectives of, patients and members of the public. They were chosen because of their long term experience of involvement in health research and their interest in impact assessment. The decision to do this review was in part due to our patient partners’ desire to quantitatively assess the impact of PPI, particularly on recruitment of patients to clinical trials, because “a trial that recruits more quickly will ultimately benefit patients more quickly.” While the review was underway, one patient partner (MO) retired and a third (RH) joined the group.

The patient partners provided input at six advisory group meetings and email correspondence in between meetings. As well as helping to decide on the review question, they helped to decide on our definition of PPI, which contextual and intervention characteristics to explore and how to categorise them, and which potential confounding factors to focus on in the risk of bias assessments. In addition to influencing these decisions, their enthusiasm and belief in the importance of this work helped to maintain the lead author’s motivation through what was a challenging piece of work. Working in partnership with patients has been a very positive experience for the researchers in the team, and we have not identified any negative effects on the research. Our current patient partners (AC and RH) report multiple positive aspects of their involvement, including being interested in the topic and endorsing its importance, feeling welcomed and respected as part of the project team, and feeling that their contributions are valued and responded to. Negative aspects have included difficulty following the conversation and contributing during teleconference meetings (sometimes necessary because of the long geographical distance between RH and the lead author) and having only a limited understanding of the mathematics of the meta-analysis.

## Results

### Characteristics of studies included in systematic review

**Fig 1:**
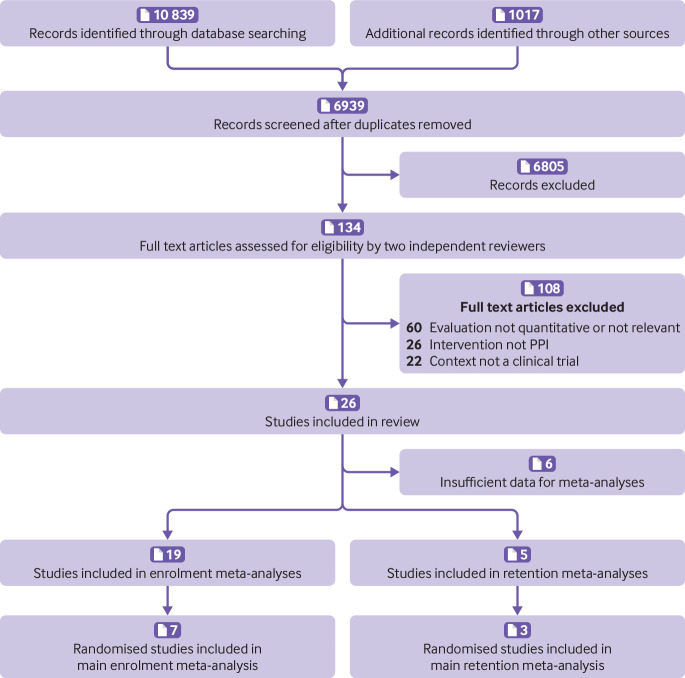
PRISMA flow diagram of records/studies included at each stage of screening and in final meta-analyses. PPI=patient and public involvement

Our search results yielded 11 856 records. After excluding duplicates, two independent reviewers screened 6939 titles and abstracts and assessed 134 full text articles for eligibility. Twenty six studies met the criteria for inclusion in the review (fig 1).

Table 3, table 4, and table 5 show the detailed characteristics of all included studies. Most were conducted in the USA or the UK, and together they covered a wide range of clinical topic areas and trial interventions. The PPI interventions were also diverse. Patients and/or members of the public were involved in different activities: eight studies involved patients or lay people in designing recruitment and retention strategies (for example, as community partners, members of a community advisory board, or focus group participants),^34^ ^41^ ^51^ ^55^ ^66^ ^69^ ^75^ ^76^ 12 studies involved patients or lay people in developing patient-facing information (for example, patient information sheets, multimedia and online interventions, recruitment advertisements, and verbal messaging),^39^ ^41^ ^43^ ^45^ ^49^ ^53^ ^58^ ^59^ ^61^ ^63^ ^66^ ^78^ and 10 studies involved patients or lay people in directly recruiting or retaining participants (for example, hiring lay/community workers or asking existing participants to refer friends/relatives).^34^ ^36^ ^46^ ^47^ ^51^ ^60^ ^65^ ^67^ ^72^ ^79^ The extent of involvement ranged from one patient advocate acting as a panellist in a one-off educational seminar for recruiting clinicians,^59^ to more than 80 people helping to develop a patient friendly online trials registry,^41^ ^42^ or community partners initiating and leading their own recruitment strategies.^51^ ^76^ Many intended purposes of involvement also existed, including increasing trust between communities and researchers,^34^ ^46^ ^51^ ^60^ ^65^ ^79^ improving the quality and acceptability of patient-facing information or recruitment messages,^39^ ^41^ ^43^ ^53^ ^61^ ^63^ ^78^ accessing potential participants via existing participants,^36^ ^67^ and increasing the cultural competence of the research among minority ethnic communities.^36^ ^46^ ^60^ ^67^ ^69^ ^72^ ^75^ ^76^ ^79^ Many of the PPI interventions also included non-PPI components, such as the involvement of other stakeholders or experts,^41^ ^58^ ^61^ ^65^ ^69^ ^75^ or novel modes of information delivery that were not a direct consequence of the PPI.^45^ ^53^ ^60^ ^67^ ^72^ ^78^ ^79^

**Table 3:** Contextual/clinical trial characteristics of studies included in review

Study	Participants	Geographical setting	Clinical trial intervention(s)/treatment(s)
Arean et al, 2003^34^ ^35^	People aged ≥65 with symptoms of depression, anxiety, and at-risk drinking	San Francisco, USA	Three types of psychosocial intervention for depression; social service model of care delivered in community geriatric medicine clinic
Chlebowski et al, 2010^36^-^38^	Healthy white men aged ≥55 years and healthy black men aged ≥50 years	USA (multisite)	Selenium and vitamin E *v* placebo for prevention of prostate cancer
Cockayne et al, 2017^39^ ^40^	People aged >65 who had attended routine podiatry appointment within previous 6 months	UK (multisite)	Podiatry intervention *v* usual care for prevention of falls in older people
Dear et al, 2012^41^ ^42^	Cancer patients consulting with their physician	Australia (multisite)	Various (multiple trials included)
Donovan et al, 2002^43^ ^44^	Men aged 50-69 years with localised prostate cancer	UK (multisite)	Surgery, radiotherapy, or monitoring for treatment of localised prostate cancer
Du et al, 2008^45^	Patients aged 21-80 years with lung cancer	Detroit, USA	Various therapeutic and non-therapeutic interventions (multiple trials included)
Ford et al, 2004^46^	African-American men aged 55-74 years	USA (multisite)	Screening for prostate, lung, and colorectal cancers
Fouad et al, 2014^47^ ^48^	Minority ethnic, low income women with low grade cervical cytological abnormalities	Jefferson County, AL, USA	Immediate colposcopy, triage, or conservative management of cytological diagnosis of atypical squamous cells of undetermined significance
Guarino et al, 2006^49^ ^50^	Gulf War veterans with fatigue, musculoskeletal pain, and/or cognitive complaints	USA (multisite)	Cognitive behavioural therapy, aerobic exercise, or both *v* usual care for treatment of Gulf War veterans’ illnesses
Horowitz et al, 2009^51^ ^52^	Adults with pre-diabetes	East Harlem, NY, USA	Community based, peer led weight loss programme to prevent diabetes
Hutchison et al, 2007^53^ ^54^	Patients with colorectal, breast, or lung cancer and clinically eligible for entry into randomised treatment trial	Glasgow, UK	Cancer treatment *v* control/standard treatment or best supportive care
Iliffe et al, 2013^55^-^57^	Patients with moderate to severe Alzheimer’s disease who had been treated with donepezil for ≥3 months	UK (multisite)	Continue donepezil, discontinue donepezil, discontinue donepezil and start memantine, or continue donepezil and start memantine, for treatment of moderate to severe Alzheimer’s disease
Kass et al, 2009^58^	Patients with cancer referred for evaluation with oncologist regarding possible participation in early phase clinical trial	USA (multisite)	Cancer treatments (various early phase clinical trials)
Kimmick et al, 2005^59^	Patients aged ≥65 years with cancer	USA (multisite)	Cancer treatments (various trials)
MacEntee et al, 2002^60^	Community dwelling older people with history of poor oral care	Vancouver, Canada	Antibacterial mouthwash to reduce tooth loss
Man et al, 2015^61^ ^62^	Adult patients with depression	UK (multisite)	12 month telehealth intervention *v* usual general practitioner care for treatment of depression
Martin et al, 2013^63^ ^64^	New mothers who self identified as black/African-American or Hispanic/Latina	New York City, USA	Behavioural educational intervention to prevent postpartum depression among black and Latina women
Moinpour et al, 2000^65^	Healthy men aged ≥55 years	USA (multisite)	Finasteride *v* placebo to prevent prostate cancer
Porter et al, 2016^66^	Patients with cancer registered at one clinical centre	Ohio, USA	Cancer treatments (various trials)
Sanders et al, 2009^67^ ^68^	Women aged ≥70 years at high risk of falls or fractures	Victoria, Australia	Vitamin D *v* placebo to prevent fractures
Tenorio et al, 2011^69^-^71^	Men and women aged 55-74 years	Denver, USA	Screening *v* routine medical care to reduce mortality from prostate, lung, colorectal, and ovarian cancers
Tenorio et al, 2014^72^-^74^	People who had smoked ≥30 pack years of cigarettes	Denver, USA	Computed tomography *v* x ray screening to diagnose and reduce mortality from lung cancer
Vicini et al, 2011^75^	Patients with cancer diagnosed and treated at one hospital	Michigan, USA	Interventions focused on cancer treatment, prevention, detection, symptom management, or cancer control (various clinical trials)
Vincent et al, 2013^76^ ^77^	Spanish speaking Latinos of Mexican origin at high risk of diabetes	Arizona, USA	Community based weight loss programme to prevent diabetes
Wallace et al, 2006^78^	Men with early stage prostate cancer	Toronto, Canada	Surgical prostatectomy *v* interstitial radiation for treatment of early stage prostate cancer
Wisdom et al, 2002^79^	African-Americans with type 2 diabetes diagnosed after age 30 years	Michigan, USA	Self management programme *v* usual care for treatment of diabetes

**Table 4:** Characteristics of patient and public involvement (PPI) interventions included in review

Study	Primary aim of intervention	PPI component(s)	Other (non-PPI) components*	Authors’ proposed mechanism
Arean et al, 2003^34^ ^35^	To improve recruitment and retention of older minority adults to trial	All recruitment and study procedures were discussed at bimonthly consumer advisory board meetings. A community member was trained by research staff to recruit and screen participants	A range of other “consumer centred” strategies including face-to-face recruitment, personalised mailings, and in-home interviews.	Overcoming stigma and mistrust barriers associated with research in minority communities
Chlebowski et al, 2010^36^-^38^	To improve rates of consent to randomisation in trial	Women already participating in a large health research project were asked to recruit their husbands	None	Women participating in clinical studies are altruistic, and their husbands share this quality and are willing to participate in a similar clinical trial
Cockayne et al, 2017^39^ ^40^	To improve trial recruitment rates	Two different PPI interventions: “bespoke user-tested” PIS: formal user testing of PIS by 30 members of public; “template developed PIS”: historical non-bespoke user testing; PPI group reviewed PIS and gave feedback.	“Bespoke user tested” PIS: design input by researchers and commercial company. “Template developed PIS”: design input by experienced researchers	Improving the quality and appearance of patient information sheets
Dear et al, 2012^41^ ^42^	To improve proportion of patients with whom participation in any clinical trial was discussed	Consumer input into design and content of consumer friendly online cancer trials registry	Online cancer trials registry developed by web company with input from staff at Australian New Zealand Clinical Trials Registry	Improving consumer knowledge and understanding of clinical trials; enabling patients to search for local trials they might like to join; providing decision support for patients considering joining a trial
Donovan et al, 2002^43^ ^44^	To improve rates of consent to randomisation in trial	In-depth interviews with potential participants who had been invited to take part	Qualitative analysis of interviews by researchers. Other qualitative research methods, including interviews with recruiters and analysis of audio recorded recruitment appointments. Findings were used to change patient information and train recruiters	Uncovering problems with information and communication during recruitment to the trial
Du et al, 2008^45^	To improve clinical trial enrolment at a large cancer centre	Presentation of a view on clinical trials from the perspectives of patients with diverse ethnic backgrounds and characteristics (in addition to standard information)	Video developed by National Cancer Institute	Positively changing patients’ knowledge of and attitudes to clinical trials
Ford et al, 2004^46^	To improve rates of recruitment to trial	Church based project sessions including consent taking, plus enhanced recruitment letter from a prominent local African-American man (arm C of trial)	Screening was conducted by African-American interviewers	Tackling four types of barriers (sociocultural, economic, individual, and study design) to recruitment of minority groups
Fouad et al, 2014^47^ ^48^	To improve rates of retention in trial and adherence to scheduled appointments	Community health advisor model, in which community members served as a link between participants and study investigators and provided additional support to participants, in addition to standard retention activities	None	Providing a trustworthy mentor to help participants overcome personal barriers to retention
Guarino et al, 2006^49^ ^50^	To improve informed consent (participants’ understanding of the trial)	Focus group of Gulf War veterans reviewed and edited PIS	None	Improving the quality and accessibility of the PIS
Horowitz et al, 2009^51^ ^52^	To increase recruitment of black and Latina people into trial	Two different PPI interventions: “public events” recruitment strategy, in which community members recruited participants at public events; “partner led” recruitment strategy, in which community advocates designed and led recruitment strategy	None	Overcoming barriers to recruitment of minority populations, including fear or mistrust of research, cultural barriers, and lack of opportunity to take part
Hutchison et al, 2007^53^ ^54^	To improve recruitment to cancer clinical trials	In addition to standard written information, patients were given access to audiovisual information designed with input from two cancer patients and presented by a local actress	Development of audiovisual patient information was led by professionals	Improving patients’ understanding of clinical trials, including randomisation
Iliffe et al, 2013^55^-^57^	To explore why, in some areas, recruitment rates had been below what was hoped	Two focus groups with patients with neurological conditions and carers, leading to changes in recruitment strategy	None	Identifying the cause of recruitment problems and suggesting remedial actions
Kass et al, 2009^58^	To improve patients’ understanding of early phase clinical trials	Intervention included video clips of five actors portraying patients who decided to enrol in a clinical trial (three) or not to enrol (two). The scripts were based on real patient narratives. The overall intervention was modified using feedback from 18 cancer patients and survivors	Intervention was a self directed, narrated, computer based presentation, including suggested questions and video clips of oncologists. Oncologists also gave feedback on the intervention	Improving patients’ understanding of the purpose and benefits of early phase clinical trials
Kimmick et al, 2005^59^	To improve recruitment of older people by physicians to cancer treatment trials	Educational intervention for physicians, including a case discussion seminar with a patient advocate panellist	The intervention also included standard information, an educational symposium, educational materials, a list of available protocols for use, and a monthly email and mail reminders for one year (with no patient input)	Enabling physicians to discuss common problems in geriatric oncology with a panel of experts
MacEntee et al, 2002^60^	To improve recruitment of ethnic minorities	At least one contact person in each community centre served as a volunteer interpreter and cultural liaison between potential recruits and researchers	Recruitment by researchers via community centres, including posters and an introductory lecture about the trial	Using active and trusted members of the community to communicate with potential recruits
Man et al, 2015^61^ ^62^	To improve recruitment to trial	PIS underwent three rounds of user testing with members of the public	Input by experts in writing for patients and graphic design (before user testing)	Improving the readability and presentation of patient information sheets
Martin et al, 2013^63^ ^64^	To improve recruitment to trial	All women who refused to participate in the trial were asked open ended questions about their reasons for refusal. The research team used this feedback to improve their recruitment message	Researchers analysed women’s feedback and made changes to the recruitment message	Identifying and overcoming barriers to recruitment
Moinpour et al, 2000^65^	To improve recruitment of minority ethnic men to the trial	“Enhanced minority recruitment programme,” included hiring African-American and Hispanic recruiters, several of whom were respected members in their minority communities	The enhanced minority recruitment programme included multiple other components such as special training in minority recruitment for site staff and consultation with experts in minority recruitment	Reducing the time taken to identify potential participants, establish trust, and introduce the trial
Porter et al, 2016^66^	To achieve a 40% increase in accrual to clinical trials over a 2 year period	The “comprehensive programme” included the leadership team informally reaching out to patients at the outset and intermittently during the campaign to increase accrual. A cancer survivor was pictured and quoted on publicity to encourage patients to enquire about clinical trial opportunities	The programme was multifaceted and included tasking centre leadership with increased oversight of the entire process of patient accrual to trials, education of all stakeholders, increased oversight of the portfolio of clinical trials by disease specific committees, and optimisation of accrual operations and infrastructure	Equipping all stakeholders (patients, their families, nurses and staff, physicians, disease specific committees, and centre leadership) with the necessary skills and information to complete the clinical trial accrual process
Sanders et al, 2009^67^ ^68^	To improve recruitment to the trial	“Word of mouth” recruitment strategy in which the research team organised morning teas for participants and invited them to bring a friend who could potentially enrol in the trial	The morning teas provided a social opportunity for participants and potential participants to meet researchers face to face	Giving participants a sense of “belonging and ownership of the project” and providing an opportunity for the friend to enrol in the trial
Tenorio et al, 2011^69^-^71^	To improve recruitment of Hispanic people to the trial	A Hispanic community focus group, including two lay people, advised on recruitment strategies	The community focus group included healthcare and research professionals. The recruitment strategy was also informed by a literature review of factors affecting recruitment of Hispanic people to clinical trials	Tailoring the recruitment plan to the Hispanic community; identifying and overcoming cultural barriers to recruitment
Tenorio et al, 2014^72^-^74^	To improve recruitment of Hispanic people to the trial	Lay consultants from the Hispanic community approached potential participants	Culturally tailored recruitment strategies including use of bilingual Hispanic staff, bilingual recruitment materials and seminars, and announcements at predominantly Hispanic churches	Overcoming cultural barriers to recruitment of Hispanic people; maximising adherence to Hispanic cultural norms
Vicini et al, 2011^75^	To decrease ethnic minority healthcare disparities and increase representation of ethnic minorities in cancer clinical trials	Minority outreach programme, involving collaboration with community based organisations from five major ethnic/minority populations. Hospital representatives worked with community leaders to develop culturally competent programmes, leading to a series of forums presented within each ethnic minority community	The collaboration included hospital representatives who were available at recruitment forums to inform patients about the clinical trials available at the hospital	Providing culture specific, bilingual cancer education and information on prevention and screening in a culturally competent manner
Vincent et al, 2013^76^ ^77^	To increase recruitment and retention in trial	Catholic church partners suggested a recruitment strategy based on healthy living/diabetes prevention presentations at the churches	None	Minimising cultural and contextual barriers to recruitment; maximising positive relationships, communication, trust, and respect, which are particularly important when working with Mexican Americans
Wallace et al, 2006^78^	To improve patients’ understanding of the treatment options and facilitate accrual to trial	During a 90 minute patient education session (intervention), a prostate cancer survivor and trial participant shared his (positive) experience of clinical trials with patients	The patient education session also included an informed consent video and a joint presentation by a urologist and radiation oncologist comparing and contrasting their modalities and introducing the concept of a randomised controlled trial	Providing balanced information about the treatment options, thereby increasing patients’ acceptance of randomisation
Wisdom et al, 2002^79^	To improve recruitment and retention in trial	Active recruitment of participants by faith based organisations and churches in the community	As well as pastors, the study’s principal investigator also made regular announcements from the pulpit	Building trust, accessibility, caring, reciprocity, and sensitivity, based on two theoretical models to improve recruitment of culturally diverse populations and access to care

PIS=patient information sheet.

* Other non-PPI components implemented before or at the same time as the PPI component. When the PPI intervention was suggested or led by PPI contributors, it was considered to be “pure” PPI even if the suggested intervention included other non-PPI aspects.

**Table 5:** Characteristics of evaluations included in review

Study	Non-PPI comparison group	Enrolment and retention outcomes assessed	Total No of participants	Evaluation design
Arean et al, 2003^34^ ^35^	“Traditional” recruitment model consisting of gatekeeper referral and media advertisements with no design input from consumers	Enrolment: proportion of potentially eligible minorities identified who were subsequently recruited to trial. Retention: proportion of minority participants completing 3 month and 6 month follow-up assessment	Enrolment: 444; retention: 95	Observational study
Chlebowski et al, 2010^36^-^38^	Mass mailing of invitation letters to potential participants	Enrolment: proportion of men targeted for recruitment who were subsequently enrolled in trial; cost per participant enrolled. Retention: not assessed	Enrolment: 60 800; retention: NA	Non-randomised controlled trial
Cockayne et al, 2017^39^ ^40^	Original PIS developed for the trial, written in accordance with the standard National Research Ethics Service template	Enrolment: proportion of participants invited who were subsequently randomised. Retention: proportion of patients retained in the trial at 3 months after randomisation	Enrolment: 6900; retention: 193	Randomised controlled trial
Dear et al, 2012^41^ ^42^	Usual approach to recruitment of trial participants, with no access to consumer friendly online trials registry	Enrolment: proportion of eligible patients consulting with a physician who subsequently self reported consent to take part in a trial. Retention: not assessed	Enrolment: 340; retention: NA	Randomised controlled trial
Donovan et al, 2002^43^ ^44^	Recruitment according to original trial protocol	Enrolment: proportion of men invited who subsequently consented to randomisation. Retention: proportion of men who consented to randomisation and subsequently accepted their allocated treatment	Enrolment: 155; retention: 108	Uncontrolled before-after study
Du et al, 2008^45^	Standard care (first visit with medical oncologist) with no access to video	Enrolment: proportion of patients who enrolled in therapeutic/non-therapeutic trials after visit with medical oncologist. Retention: not assessed	Enrolment: 126; retention: NA	Randomised controlled trial
Ford et al, 2004^46^	Standard trial recruitment procedures at health site; consent taken by mail; screening conducted by African-American and white interviewers (arm D of trial)	Enrolment: proportion of men contacted and found eligible who were randomised to trial. Retention: not assessed	Enrolment: 6246; retention: NA	Randomised controlled trial
Fouad et al, 2014^47^ ^48^	Standard retention activities (reminder calls, cards, and incentives)	Enrolment: not assessed. Retention: proportion of participants who attended all follow-up visits	Enrolment: NA; retention: 632	Randomised controlled trial
Guarino et al, 2006^49^ ^50^	Original PIS designed by researchers	Enrolment: proportion of patients invited who subsequently refused to take part in trial. Retention: proportion of participants missing any primary outcome data	Enrolment: 2793; retention: 1092	Randomised controlled trial
Horowitz et al, 2009^51^ ^52^	Other recruitment strategies: clinical referral, special recruitment events, and recruitment via community based organisations	Enrolment: proportion of people approached who were subsequently enrolled in the trial. Retention: not assessed	Enrolment: 554; retention: NA	Observational study
Hutchison et al, 2007^53^ ^54^	Standard trial specific written patient information	Enrolment: proportion of patients invited who were subsequently enrolled into a trial. Retention: not assessed	Enrolment: 173; retention: NA	Randomised controlled trial
Iliffe et al, 2013^55^-^57^	Original recruitment strategy before focus groups	Enrolment: proportion of total participants (all regions) recruited in intervention exposed regions before versus after intervention. Retention: not assessed	Enrolment: 200; retention: NA	Controlled before-after study
Kass et al, 2009^58^	Informational pamphlet developed by the National Cancer Institute called “Taking part in clinical trials: what cancer patients need to know”	Enrolment: proportion of patients invited to take part in a clinical trial who subsequently decided to enrol in the trial (self reported). Retention: not assessed	Enrolment: 130; retention: NA	Randomised controlled trial
Kimmick et al, 2005^59^	Standard information only (periodic notification of all existing trials and website access)	Enrolment: proportion of older cancer patients registered who were subsequently accrued to a cancer treatment trial. Retention: not assessed	Enrolment: 3032; retention: NA	Randomised controlled trial
MacEntee et al, 2002^60^	Announcements in newspapers to attract potential recruits	Enrolment: proportion of initial responders who were subsequently recruited to the trial; cost per recruit. Retention: not assessed	Enrolment: 887; retention:: NA	Observational study
Man et al, 2015^61^ ^62^	Standard information sheet designed by researchers using National Research Ethics Service guidelines	Enrolment: proportion of patients who received PIS and were subsequently randomised to trial. Retention: not assessed	Enrolment: 1364; retention: NA	Randomised controlled trial
Martin et al, 2013^63^ ^64^	Original recruitment message (before intervention)	Enrolment: proportion of women approached who were subsequently randomised to trial. Retention: not assessed	Enrolment: 668; retention: NA	Uncontrolled time series
Moinpour et al, 2000^65^	Original minority recruitment protocol (before enhanced programme introduced)	Enrolment: proportion of total participants (all ethnicities) who were from ethnic minorities. Retention: not assessed	Enrolment: 18 882; retention: NA	Uncontrolled before-after study
Porter et al, 2016^66^	Original clinical trials accrual programme (before comprehensive programme introduced)	Enrolment: annual number of patient accruals, accruals per active trial, and accrual rate (number of patients accrued in a given calendar year divided by number of new analytical cases seen at the cancer centre for that same year). Retention: not assessed	Enrolment: 35 853; retention: NA	Uncontrolled time series
Sanders et al, 2009^67^ ^68^	“Targeted mail out” recruitment strategy consisting of postal invitations to women aged ≥70 years listed on government agency databases	Enrolment: proportion of people invited who were subsequently enrolled in the trial. Retention: not assessed	Enrolment: 21 600; retention: NA	Observational study
Tenorio et al, 2011^69^-^71^	Recruitment plan for general population	Enrolment: proportion of total participants (all ethnicities) who were Hispanic before versus after intervention. Retention: not assessed.	Enrolment: 21 162; retention: NA	Controlled before-after study
Tenorio et al, 2014^72^-^74^	Recruitment plan for general population	Enrolment: proportion of total participants (all ethnicities) who were Hispanic in regions exposed and not exposed to the intervention. Retention: not assessed	Enrolment: 53 053; retention: NA	Non-randomised controlled trial
Vicini et al, 2011^75^	Clinical trial accrual process before introduction of the minority outreach programme	Enrolment: annual number of minority patients accrued, and as a proportion of total patients accrued. Retention: not assessed	Enrolment: 3056; retention: NA	Uncontrolled time series
Vincent et al, 2013^76^ ^77^	Other recruitment strategies: flyers, posters, and email announcements; community events; health provider referrals	Enrolment: proportion of people approached/referred who were subsequently enrolled in trial. Retention: not assessed	Enrolment: 279; retention: NA	Observational study
Wallace et al, 2006^78^	Eligible patients were individually approached by a clinical research associate and invited to view the informed consent video	Enrolment: proportion of patients attending educational session (intervention) or watching informed consent video (comparator) who subsequently consented to randomisation Retention: not assessed	Enrolment: 290-324 (exact figure unknown owing to data discrepancies); retention: NA	Uncontrolled before-after study
Wisdom et al, 2002^79^	Recruitment from local healthcare system (via mail)	Enrolment: proportion of patients contacted who subsequently enrolled in the trial. The denominator used for the PPI exposed group was the estimated number of faith based organisation participants with diabetes, as the comparator intervention (recruitment via health system) targeted only patients with diabetes. Retention: proportion of participants who attended all seven intervention sessions	Enrolment: 1177; retention: 102	Observational study

NA=not applicable; PPI=patient and public involvement; PIS=patient information sheet.

### Characteristics of studies included in meta-analyses

We included 19 studies (21 PPI interventions) reporting data from 178 921 participants in our enrolment meta-analyses and five studies (six PPI interventions) reporting data from 6520 participants in our retention meta-analyses. Table 6 shows the aggregate characteristics of these studies, including those used in subgroup and sensitivity analyses.

**Table 6:** Aggregate characteristics of studies included in meta-analyses. Values are numbers of studies with specified characteristic unless stated otherwise

Characteristic	Enrolment meta-analysis (n=19)	Retention meta-analysis (n=5)
**Evaluation features**		
No of people included	Range 126-60 800 (median 887)	Range 95-4599 (median 632)
Year of publication	Range 2002-17 (median 2009)	Range 2002-17 (median 2006)
Study design:		
Randomised	7	3
Non-randomised	12	2
No of PPI interventions evaluated:		
One	17	4
Two	2	1
Enrolment rate denominator:		
Pre-eligibility screening	12	NA
Post-eligibility screening	6	NA
Unknown	1	NA
Risk of bias*:		
Low	4	3
Some concerns	2	0
High/serious	12	1
Critical	1	1
**Context**		
Geographical setting:		
Australia	2	0
Canada	1	0
UK	5	1
USA	11	4
Clinical trial intervention type:		
Simple	7	0
Complex	9	5
Mixed/both	3	0
Clinical trial recruitment setting:		
Healthcare	9	2
Community	3	1
Mixed/both	8	2
PPI in choosing research question/topic (context)	3	0
**PPI intervention features**		
PPI activity:		
Recruitment/retention strategies	6	1
Patient-facing information	9	2
Direct recruitment/retention	9	3
PPI intervention was chosen/designed specifically to increase recruitment or retention	18	3
PPI model:		
One-off	10	3
Intermittent	3	1
Full team membership	6	1
No of PPI contributors involved:		
One or two	1	1
Three or more	18	5
Unknown	1	0
PPI contributor(s) had lived experience of condition under study	12	0
PPI was visible to potential trial participants	11	3
Intervention included some non-PPI components	14	3
PPI was formal qualitative research	1	0
**Findings**		
Impact of PPI intervention on outcome (enrolment/retention rate) relative to comparator:		
Significantly higher enrolment/retention	11	1
No significant difference in enrolment/retention	8	4
Significantly lower in enrolment/retention	1	0

NA=not applicable; PPI=patient and public involvement.

* For randomised studies, the following levels are possible: low, some concerns, high; for non-randomised studies, the following levels are possible: low, moderate, serious, critical. These differences are due to differences in tools used to assess risk of bias.

Six studies could not be included in the enrolment meta-analyses owing to insufficient data, despite our attempts to contact study authors and identify related papers. Three of these studies reported no significant impact of PPI interventions on enrolment,^58^ ^59^ ^65^ and the other three studies reported an increase in enrolment rates associated with PPI interventions (statistical significance unknown).^66^ ^75^ ^78^

### Risk of bias of studies included in meta-analyses

Of the eight randomised studies, only one was deemed to be at “high” risk of bias owing to missing outcome data,^41^ two had “some concerns,”^45^ ^46^ and five had “low” risk of bias.^39^ ^47^ ^49^ ^53^ ^61^ Of the 12 non-randomised studies, 11 were deemed to be at “serious” risk of bias,^36^ ^43^ ^51^ ^55^ ^60^ ^63^ ^67^ ^69^ ^72^ ^76^ ^79^ and one was deemed to be at “critical” risk of bias owing to potential uncontrolled confounding by patient population, time, or both.^34^ Often this was because the study was opportunistic—for example, comparing the success of different recruitment strategies—rather than designed specifically to evaluate the impact of PPI versus non-PPI on enrolment or retention.

### Impact of PPI interventions on enrolment

#### Individual study findings

Half (11/21) of the PPI interventions included in our meta-analysis were associated with significantly higher enrolment rates compared with no PPI or non-PPI interventions,^36^ ^43^ ^46^ ^51^ ^55^ ^60^ ^63^ ^67^ ^69^ ^72^ ^76^ nine PPI interventions were not significantly associated with enrolment rate,^35^ ^39^ ^41^ ^45^ ^49^ ^51^ ^53^ ^61^ and one PPI intervention was associated with significantly lower enrolment (odds ratio 0.41, 95% confidence interval 0.23 to 0.72).^79^ In this study, lay community members (faith based organisations) attempted to directly recruit African-Americans with diabetes to the trial; however, this yielded a lower enrolment rate than recruitment via the health system (non-PPI). The authors stated that this was not surprising, given “the nature of the provider-patient relationship” and because “African Americans may be less inclined to have their personal health history known by other members of their church congregation, given the stigma associated with chronic illnesses.”^79^ Contrast this with Vincent et al’s study, which showed the largest PPI effect size in our sample (odds ratio 13.48, 6.07 to 29.95): here, lay community members (Catholic church partners, some of whom shared a high risk of diabetes with the Mexican-American target population) initiated, co-designed, and co-delivered a recruitment strategy that was highly successful compared with strategies initiated by the researchers.^77^ (Note, however, that both of these outlying studies were non-randomised and judged to be at high risk of bias.)

#### Main meta-analysis (randomised studies only)

**Fig 2:**
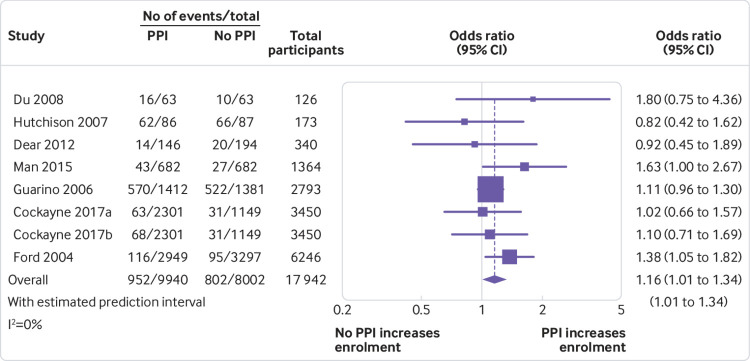
Odds ratios for patient enrolment in clinical trial with versus without patient and public involvement (PPI) intervention (randomised studies only)

We included seven randomised studies (eight PPI interventions) in our main meta-analysis. These interventions all consisted of patient or lay involvement in the design or delivery of patient information, with Ford et al’s intervention also including recruitment sessions hosted by churches in the target community.^46^ Pooling the data from seven randomised studies in our main meta-analysis showed that, on average, PPI interventions modestly but significantly increased the odds of a patient enrolling in a clinical trial compared with no PPI (odds ratio 1.16, 1.01 to 1.34; P=0.04). We found low heterogeneity between studies (I^2^=0.0%), yielding a 95% prediction interval of the odds ratio of 1.01 to 1.34 (fig 2).

#### Secondary meta-analysis and subgroup analyses (randomised and non-randomised studies combined)

**Fig 3:**
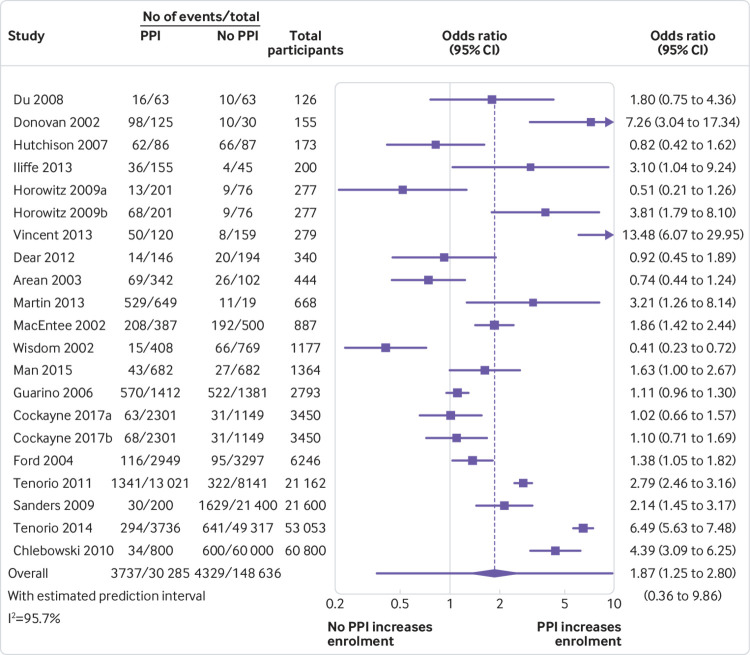
Odds ratios for patient enrolment in clinical trial with patient and public involvement (PPI) intervention versus no PPI or non-PPI intervention (randomised and non-randomised studies combined)

Our secondary meta-analysis, combining 19 randomised and non-randomised studies (21 PPI interventions), also found that, on average, PPI interventions significantly increased the odds of a patient enrolling in a clinical trial compared with no PPI or non-PPI interventions (odds ratio 1.87, 1.25 to 2.80; P=0.004). We found substantial heterogeneity between studies (I^2^=95.7%), yielding a 95% prediction interval of the odds ratio of 0.36 to 9.86 (fig 3). Exploratory subgroup analyses showed that the overall positive association between PPI interventions and enrolment substantially increased when at least one involved person had lived experience of the health condition under study (odds ratio 3.14, 1.89 to 5.22) and all but disappeared when the involved people had no such lived experience (1.07, 0.74 to 1.53). Meta-regression confirmed that this effect was statistically significant (P=0.02). Subgroup differences between any of the other variables explored (appendix 2), including trial intervention type (simple versus complex), the timing of involvement (designing recruitment and retention strategies versus developing patient-facing information versus direct recruitment or retention of participants), and enrolment rate denominator (before versus after eligibility screening), were not found to be statistically significant using meta-regression (P>0.3). Meta-regression was not able to explain the high between study heterogeneity, but it may be due in part to the diverse range of evaluation methods used and the high risk of bias by confounding in non-randomised studies. It could also be explained by heterogeneity of the PPI interventions: almost all of the PPI interventions in the high quality, randomised studies were aimed at improving patient information, whereas the more complex and more unusual interventions were largely evaluated using poorer quality observational or quasi-experimental methods.

#### Sensitivity analyses and Peters’ test

The positive overall association between PPI interventions and enrolment remained statistically significant throughout all sensitivity analyses except when we excluded interventions with non-PPI components from the secondary analysis (see appendix 3). Although the estimated effect of PPI interventions actually increased in this analysis (odds ratio=2.70), the exclusion of 15/21 studies yielded a very wide 95% confidence interval (0.83 to 8.84). We could not restrict this particular sensitivity analysis to randomised studies because this subsample included only one “pure” PPI intervention.^49^ Peters’ test showed no evidence of bias due to small study effects (P=0.92 for main analysis; P=0.59 for secondary analysis).

#### Cost effectiveness of PPI interventions

Of the two studies reporting the cost per participant enrolled, MacEntee et al reported that a PPI strategy to recruit participants at community centres through a local contact person, although more effective, was more than twice the cost per participant of a non-PPI strategy that used postal invitations ($23 (£18; €20) *v* $11).^60^ Chlebowski et al reported that a PPI strategy to recruit trial participants via existing research participants was only one quarter the cost of a non-PPI strategy that used commercial mailing lists to send postal invitations ($59 *v* $259 per participant enrolled).^36^

### Impact of PPI interventions on retention

#### Main meta-analysis (randomised studies only)

Pooling the data from three randomised studies (four PPI interventions) in our main meta-analysis did not show a statistically significant effect of PPI interventions on participant retention (odds ratio 1.16, 0.33 to 4.14; P=0.73). Results varied widely across studies, with effect estimates ranging from odds ratios of 0.38 to 2.52 (I^2^=83.5%; 95% prediction interval 0.06 to 22.37; appendix 4).

#### Secondary meta-analysis (randomised and non-randomised studies combined)

Our secondary meta-analysis, combining five randomised and non-randomised studies (six PPI interventions), also found no statistically significant effect of PPI interventions on participant retention, compared with no PPI or non-PPI interventions (odds ratio 1.20, 0.52 to 2.77; P=0.59). Again, we found substantial heterogeneity between studies (I^2^=78.3%), yielding a 95% prediction interval of the odds ratio of 0.20 to 7.18 (forest plot in appendix 5). At the individual study level, only one PPI intervention was significantly associated with retention: this constituted use of lay community health advisers to support participants (the only PPI intervention specifically targeting retention), leading to a significant improvement in retention rates (odds ratio 2.52, 1.82 to 3.50).^47^ Apart from this example, the PPI interventions primarily targeted enrolment, not retention. We did not do subgroup analyses for retention outcomes because of the small sample size.

#### Sensitivity analyses and Peters’ test

Sensitivity analyses did not alter the findings (appendix 6), and Peters’ test showed no evidence of bias due to small study effects (P=0.44 for main analysis; P=0.41 for secondary analysis).

## Discussion

This review identified a variety of PPI interventions aimed at improving enrolment and retention of participants in clinical trials. Patients and lay members of the public were involved in designing recruitment and retention strategies and patient-facing information, identifying and approaching potential participants, and troubleshooting when recruitment was poor. We did not identify any studies that assessed the impact on enrolment or retention of PPI in developing the trial question or designing the trial itself.

On average, PPI interventions significantly increased the odds of a patient enrolling in a clinical trial, relative to no PPI or non-PPI recruitment interventions. This remained statistically significant regardless of whether non-randomised studies were excluded or included, as well as in sensitivity analysis that removed studies at highest risk of bias. To illustrate what our main findings could mean in practice: in a hypothetical sample of 1000 patients, of which typically 100 enrol (consistent with the 10% average enrolment rate in our sample of studies), a PPI intervention similar to those included in our meta-analysis of randomised studies would likely lead to between one and 30 (average 14) extra patients being enrolled. As these PPI interventions were mostly restricted to patient or lay involvement in the design or delivery of patient information, the effect size might be even larger for PPI that begins at earlier stages of trial design, as the opportunity to influence patients’ views and experiences would extend beyond just the provision of information.

A key exploratory finding was that the effect size was significantly greater when the people involved had lived experience of the health condition under study, compared with no such lived experience. This is consistent with the view that patients and carers can benefit research through their role as “expert in lived experience,”^80^ although the precise mechanisms linking such expertise with improvements in enrolment and retention are unclear—something that we are exploring in a complementary realist analysis of the included studies. This finding, along with all other subgroup analysis and meta-regression findings, should be interpreted with caution owing to the potential for study level confounding.

Far fewer studies evaluated the impact of PPI interventions on retention of trial participants. They showed, on average, a modest but non-significant improvement in retention; the very wide 95% confidence intervals mean that we cannot rule out a potentially large increase or decrease in retention associated with PPI. None of the PPI interventions in the retention analysis included people with lived experience of the health condition under study, and most of them primarily targeted enrolment rather than retention.

### Strengths and limitations of review

To our knowledge, this is the first attempt to combine data on the impact of PPI on enrolment and retention in health research, providing a quantitative summary and exploring the influence of contextual and intervention factors. Our results are consistent with previous observational studies that suggested an average positive association between PPI and success of recruitment in UK based health studies.^15^ ^16^ Unlike these previous studies, our review encompassed all geographies and clinical areas, and we were able to explore, to some extent, the influence of the characteristics and context of PPI.

Our review has several limitations. Most of the interventions included non-PPI components, and we could not separate out the effects of these from the effects of the PPI components. When interventions including non-PPI components were excluded in a sensitivity analysis of both randomised and non-randomised studies combined, PPI was still associated with improved enrolment but with reduced certainty due to the decrease in sample size.

We were unable to explore the influence of many potentially important factors such as underlying programme theory, the fidelity and sustainability of interventions, the quality of relationships between involved patients and researchers, and the attitude of research leaders towards PPI.^23^ ^81^ We are undertaking a realist analysis of the included papers to shed more light on these complexities.^23^ The framing of PPI as a complex intervention is itself controversial,^82^ but we believe that this approach, alongside a range of other perspectives, can usefully contribute to the much broader debate about the impact of PPI in health research.

Our 95% prediction intervals should be interpreted with caution because prediction intervals have been reported to be less reliable in meta-analyses with unbalanced study sizes.^83^ Also, we were unable to provide a useful summary of the cost effectiveness of PPI, because very few studies included an economic impact assessment; thus an “effective” PPI intervention may not necessarily be cost effective. However, financial modelling of the impact of PPI in a typical oncology trial suggests that PPI interventions that improve enrolment may add considerable financial value.^84^

Finally, the findings of this study say nothing about the quality or ethical acceptability of PPI in the included studies or patients’ views on the importance of the clinical trials being conducted. PPI may improve enrolment, but this does not rule out negative effects such as an emotional cost to the people involved or patients feeling coerced into enrolling.^85^ Should patients assume that all trials are conducted for their benefit and automatically endorse every trial? Do (and should) involved patients have the necessary skills to assess the risks involved on behalf of their fellow patients? These are important dilemmas that are beyond the scope of this study.

### Implications for clinical trialists and PPI policy makers

Our findings add support to the case for involving patients and carers in the design and conduct of clinical trials. In the UK, funding proposals and protocols for trials are often reviewed by institutional lay panels; our review suggests that, ideally, at least some of these reviewers would be patients and carers with lived experience of the health condition under study.

The apparent failure of some PPI interventions to improve enrolment and retention shows that many factors other than PPI also influence these outcomes. In addition, some PPI interventions in our review were one of several recruitment strategies used by clinical trialists and may not have been sufficient alone; for example, Sanders et al found that although their word of mouth PPI strategy was relatively effective at enrolling those it reached, it contributed only 2.2% of the total participants owing to limited reach (200 people), compared with 70.3% for the targeted mail-out strategy (which reached 21 400 people).^67^ PPI will not solve all recruitment and retention problems, and clinical trialists would be wise to implement multiple additional strategies to minimise the risk of poor enrolment and retention. Furthermore, involving patients in the early stages of trial development can sometimes lead researchers to abandon the whole idea of the trial,^86^ suggesting that if the target patients are not convinced that the trial question is worth answering, PPI in later stages of the trial (such as those seen in this review) may be futile.

### Unanswered questions and future research

Well planned, high quality evaluations are needed to improve our understanding of the impact of PPI on enrolment and retention in clinical trials. In particular: which types of PPI work best in particular settings and contexts; the mechanisms underlying the impact of PPI on enrolment and retention; the cost effectiveness of PPI interventions (an important part of the drive to improve trial efficiency); the impact of PPI interventions specifically targeting retention (which has received very little attention relative to enrolment); and the impact of PPI at the early stages of trial proposal and design.

**What is already known on this topic**

Patient and public involvement (PPI) in clinical trials has the potential to improve rates of enrolment and retention of participants
PPI may help by improving trial design, optimising recruitment and retention strategies and patient-facing materials, or directly approaching potential participants
Whether, when, or by how much, PPI affects rates of enrolment and retention of participants is not known

**What this study adds**

The nature of PPI interventions and the impact of these on trial enrolment and retention vary widely between studies
On average, PPI interventions seem to modestly but significantly increase the odds of participant enrolment
The impact of PPI on retention rates is less clear and requires further primary research evaluating PPI interventions that specifically target retention

## Web Extra

Extra material supplied by the author

## Notes

Contributors: JCC, AC, SPZ, DE, and SR conceived and designed this review. JCC, IRC, and AP undertook searches, record screening, and data extraction (supervised by JCC). JH wrote the code for and ran the meta-analyses in Stata. All authors contributed to interpretation of the results. JCC wrote the manuscript, and all authors commented on the draft and approved the final version. The corresponding author attests that all listed authors meet authorship criteria and that no others meeting the criteria have been omitted. JCC is the guarantor.

Ethical approval: Not needed.

Data sharing: The dataset is available on request from the corresponding author.

Transparency declaration: The lead author (study guarantor) affirms that this manuscript is an honest, accurate, and transparent account of the study being reported; that no important aspects of the study have been omitted; and that any discrepancies from the study as planned (and, if relevant, registered) have been explained.
