# Supplementary material for: Impact of patient and public involvement on enrolment and retention in clinical trials: systematic review and meta-analysis
Source: BMJ. 2018 Nov 28;363:k4738. doi: 10.1136/bmj.k4738 (PMC6259046; doi:10.1136/bmj.k4738)
Supplement: Supplementary file 1 — Appendix 1-6 [file croj045186.ww1.pdf]

Appendix 1: Search strategy

	**Search domain**	**Search terms**
1	Clinical trials	trial*.mp OR exp Clinical Trial as Topic/
2	PPI & recruitment/retention, with focus on PPI	((consumer? or citizen? or client? or carer? or communit? or lay or patient? or public? or service user? or survivor? or stakeholder? or family or families or relative? or parent?) AND (involv* or collaborat* or engage* or partner* or consult* or advis* or emancipat* or empower* or advocat* or embed* or represent*) OR community-based OR participatory).ti OR *Consumer Participation/ OR *Patient Participation/ OR *Community-Based Participatory Research/<br>**AND**<br>Patient Selection/ OR exp Informed Consent/ OR Research design/ OR Patient Dropouts/ OR enrol*.ab./freq=2 OR recruit*.ab./freq=2 OR participat*.ab./freq=2 OR enlist*.ab./freq=2 OR consent*.ab/freq=2 OR refus*.ab/freq=2 OR accru*.ab/freq=2 OR retention.ab/freq=2 OR attrition.ab/freq=2 OR followup.ab/freq=2 OR follow-up.ab/freq=2 OR dropout*.ab/freq=2 OR drop-out*.ab/freq=2 OR withdr*.ab/freq=2
3	PPI & recruitment/retention, with focus on recruitment/retention	((consumer? or citizen? or client? or carer? or communit* or lay or patient? or public? or service user? or survivor? or stakeholder? or family or families or relative? or parent?) adj3 (involv* or collaborat* or engage* or partner* or consult* or advis* or emancipat* or empower* or advocat* or embed* or represent*) or community-based or participatory).ab,ti OR Consumer Participation/ OR Patient Participation/ OR Community-Based Participatory Research/<br>**AND**<br>*Patient Selection/ OR *Informed Consent/ OR *Informed Consent By Minors OR *Research design/ OR *Patient Dropouts/ OR (enrol* OR recruit* OR participat* OR enlist* OR consent* OR refus* OR accru* OR retention OR attrition OR followup OR follow-up OR dropout* OR drop-out* OR withdr*).ti
4	2 or 3	
5	PPI outcomes	(impact* or effect* or adapt* or modif* or change* or develop* or design* improve* or worse* or increase* or boost* or decreas* or reduc* or differ* or edit* or suggest*).ab,ti
6	1 and 4 and 5	

Appendix 2: Forest plots showing subgroup analyses for enrolment outcome

(a) Enrolment rate denominator (pre vs. post eligibility screening)

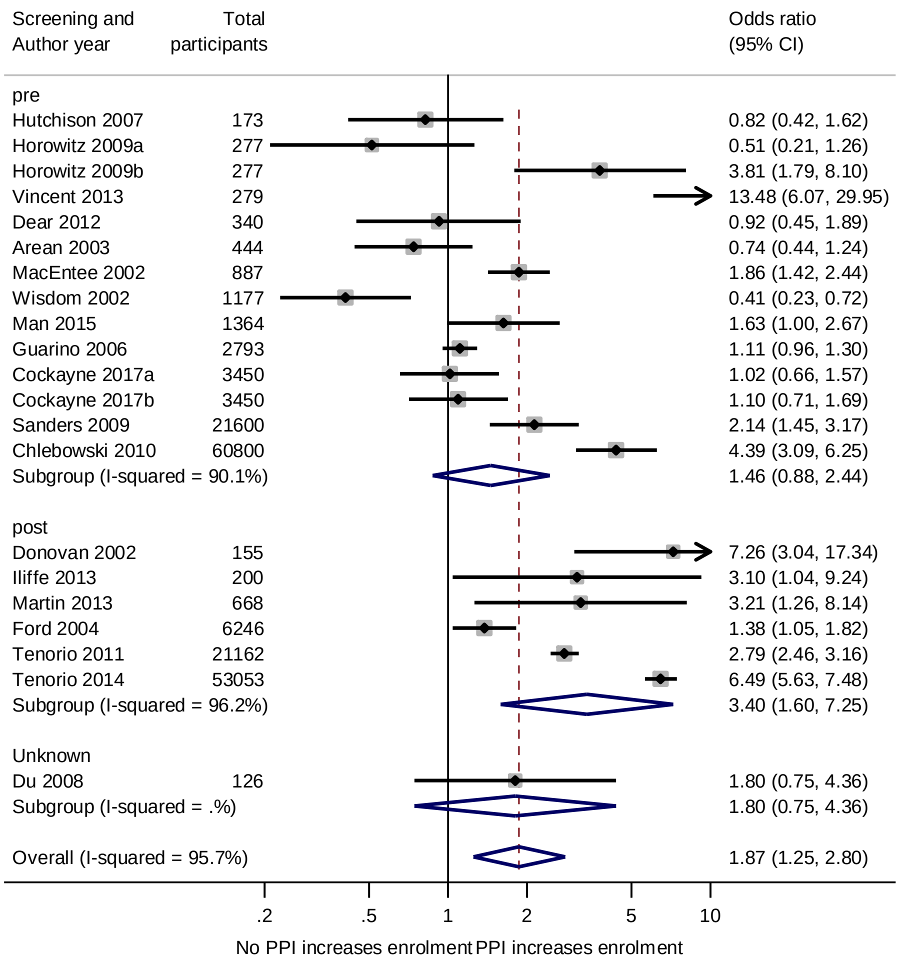

(b) Trial recruitment setting (context)

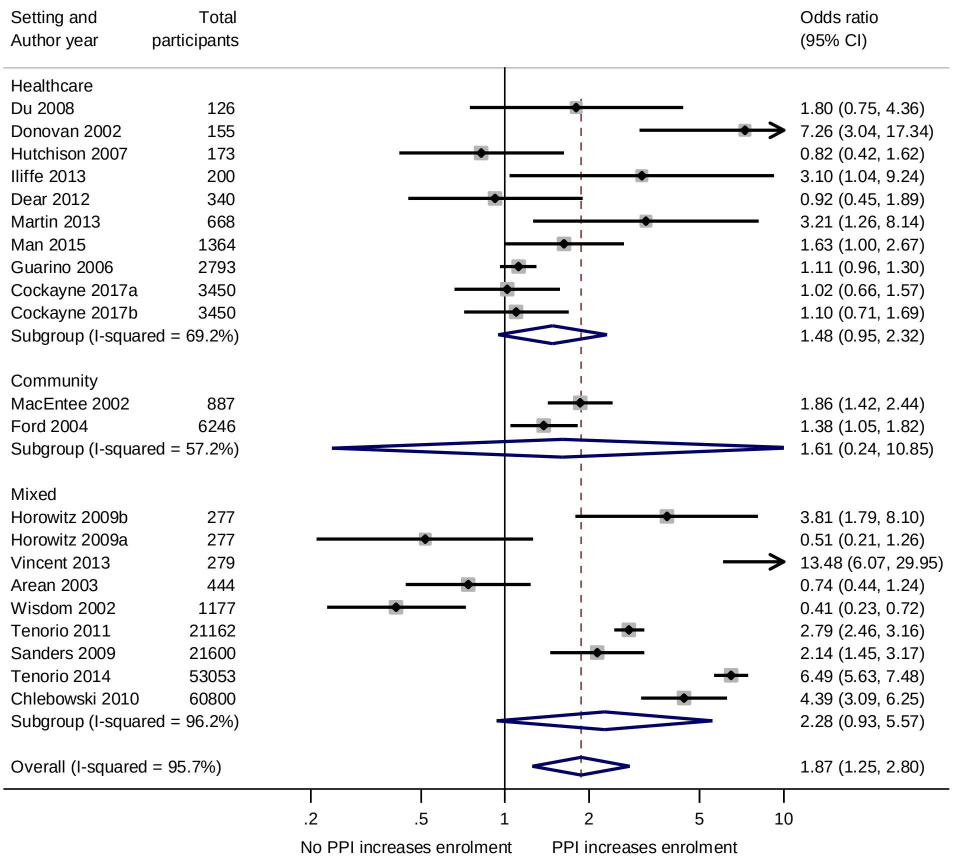

(c) Trial intervention type (context)

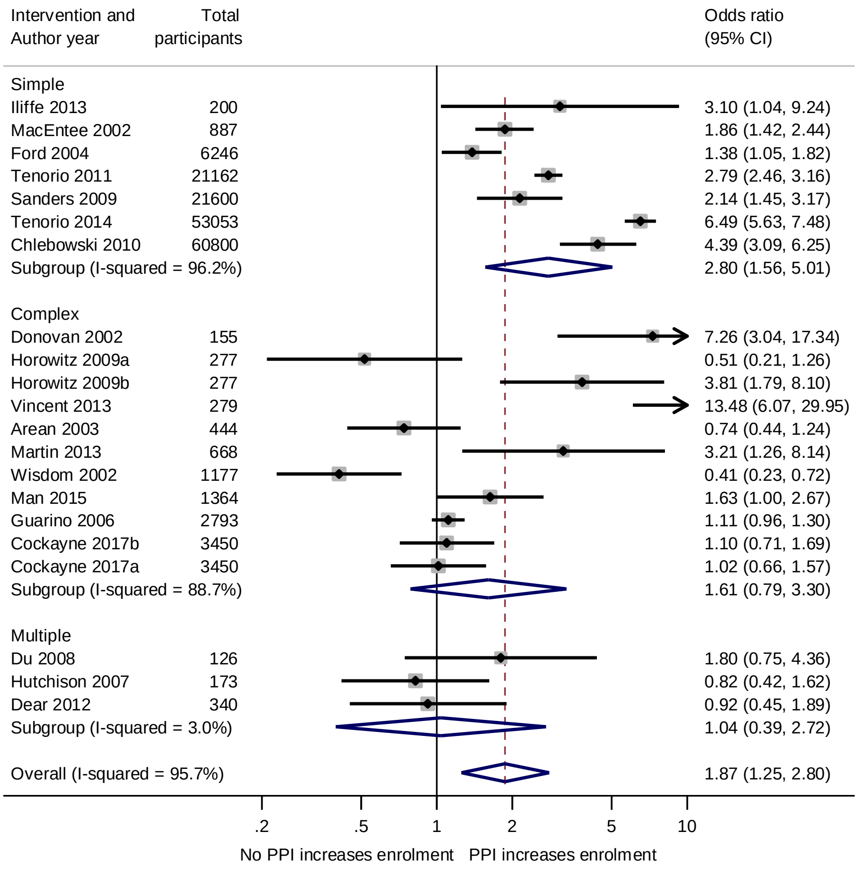

(d) PPI in choosing research question/topic (context)

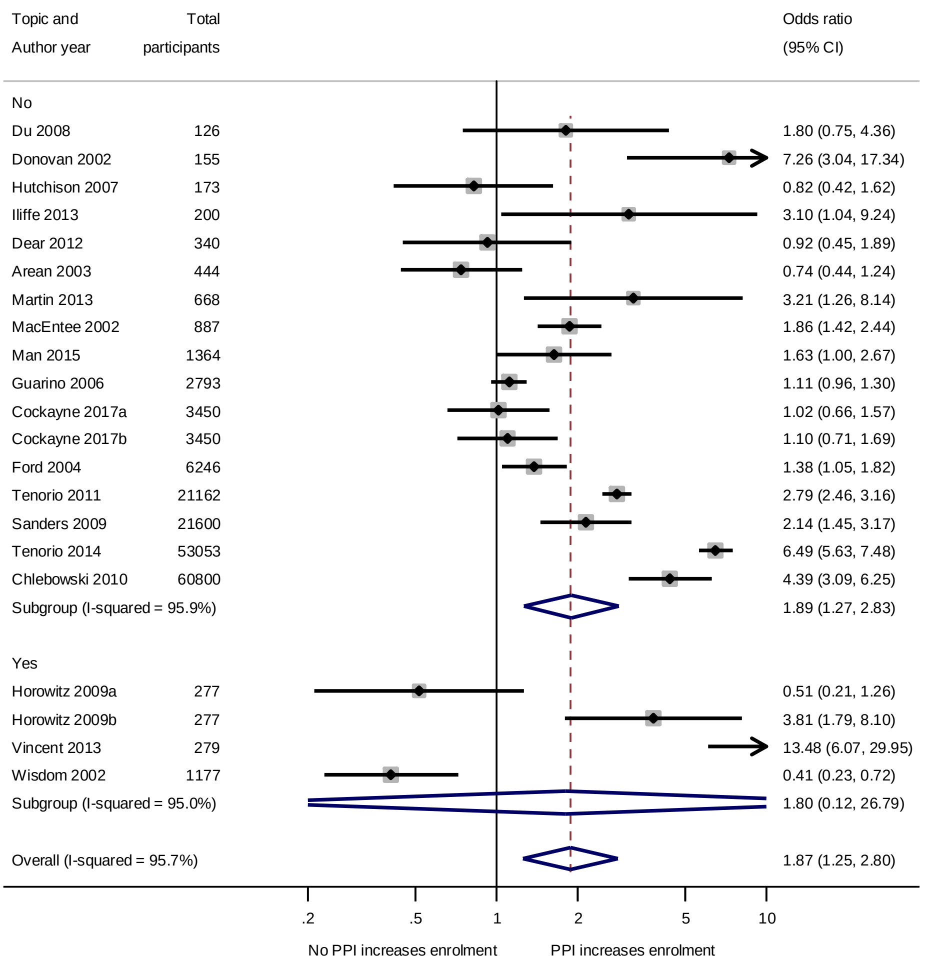

(e) Timing/activity of PPI intervention

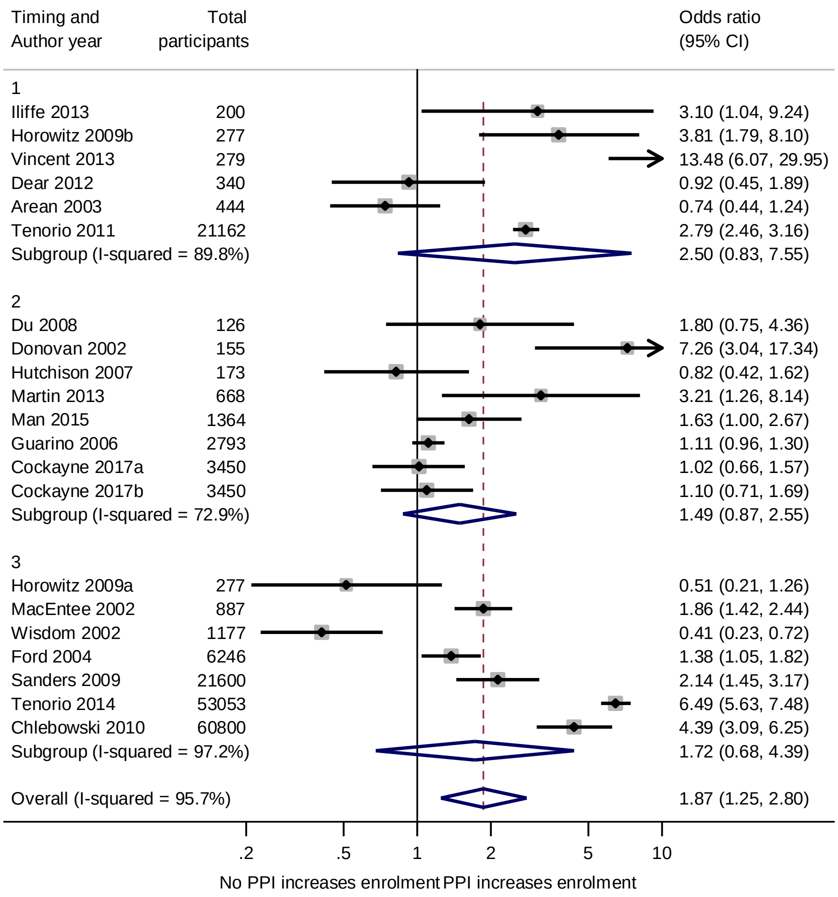

Key:

1 = designing recruitment or retention strategy

2 = developing patient-facing information

3 = directly approaching / recruiting or retaining participants

(f) Number of the activities (e) targeted by PPI intervention

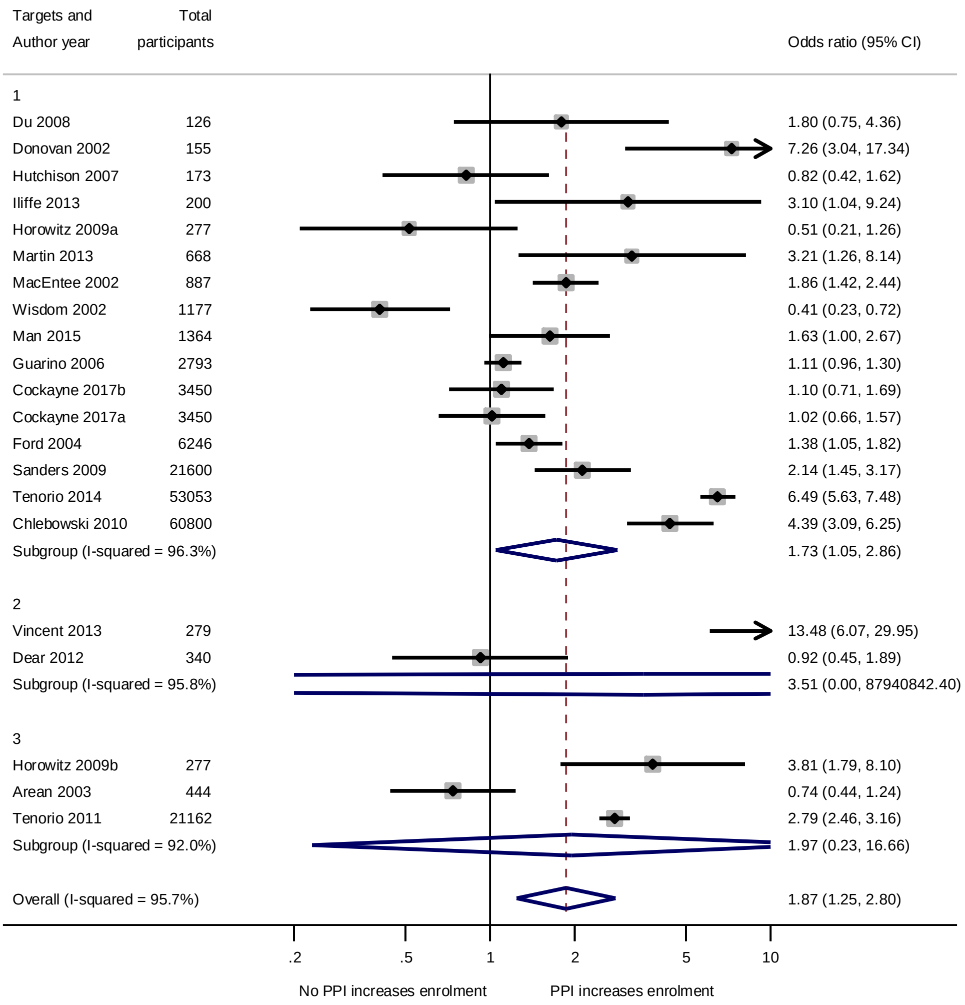

(g) PPI intervention chosen/designed specifically to increase recruitment or retention

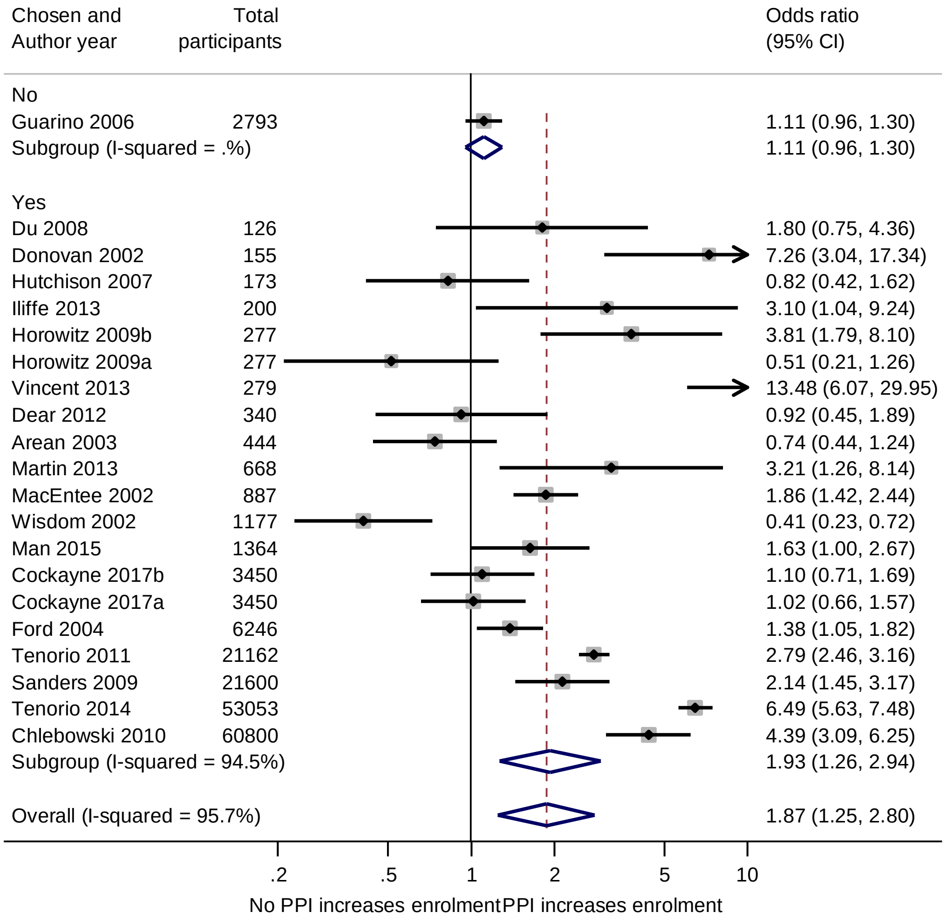

(h) PPI model

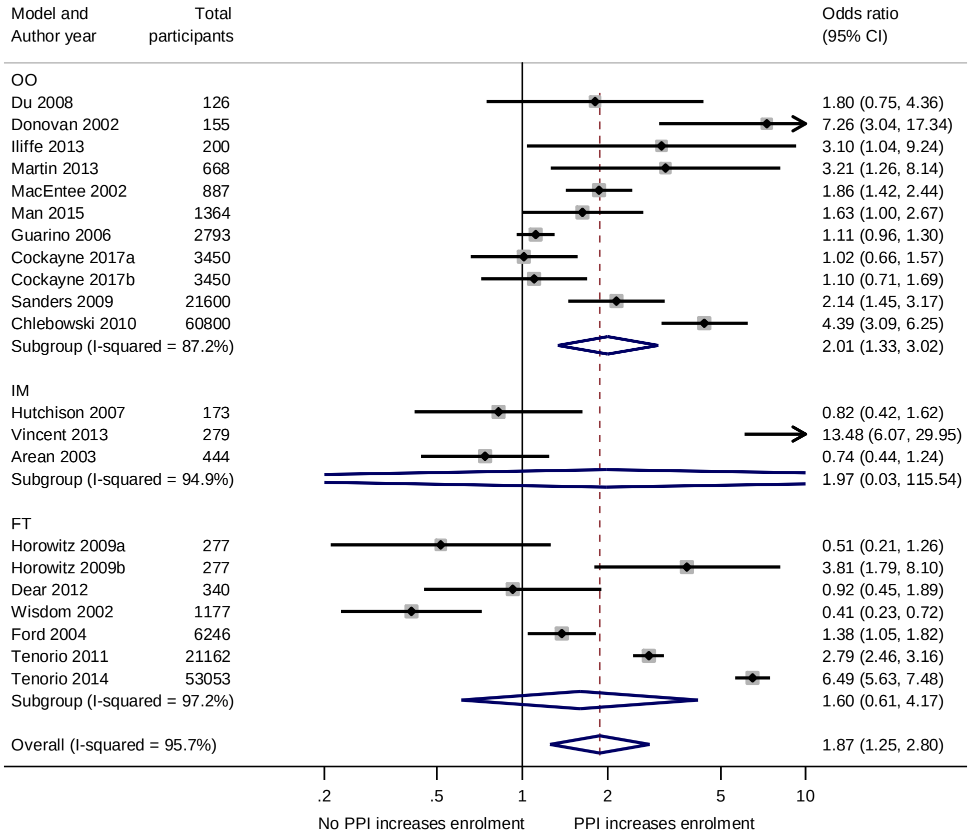

Key:

OO = One-off

IM = Intermittent

FT = Full team membership

(i) Number of PPI contributors involved

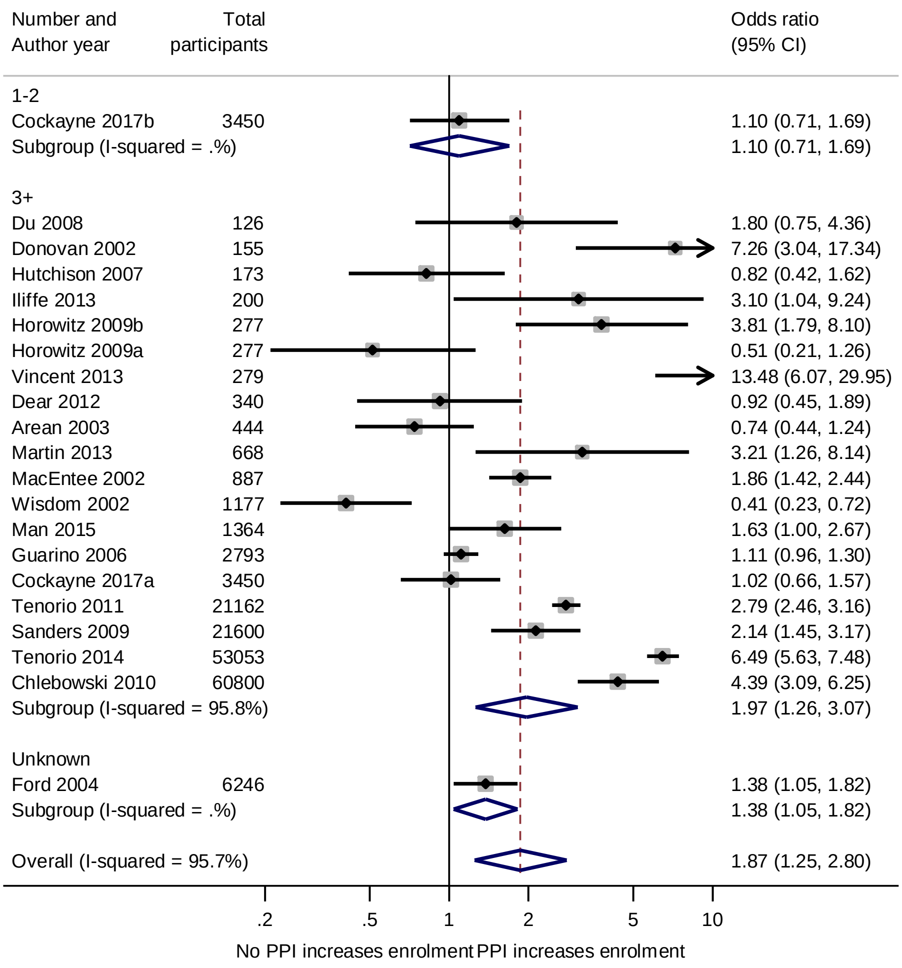

(j) Lived experience

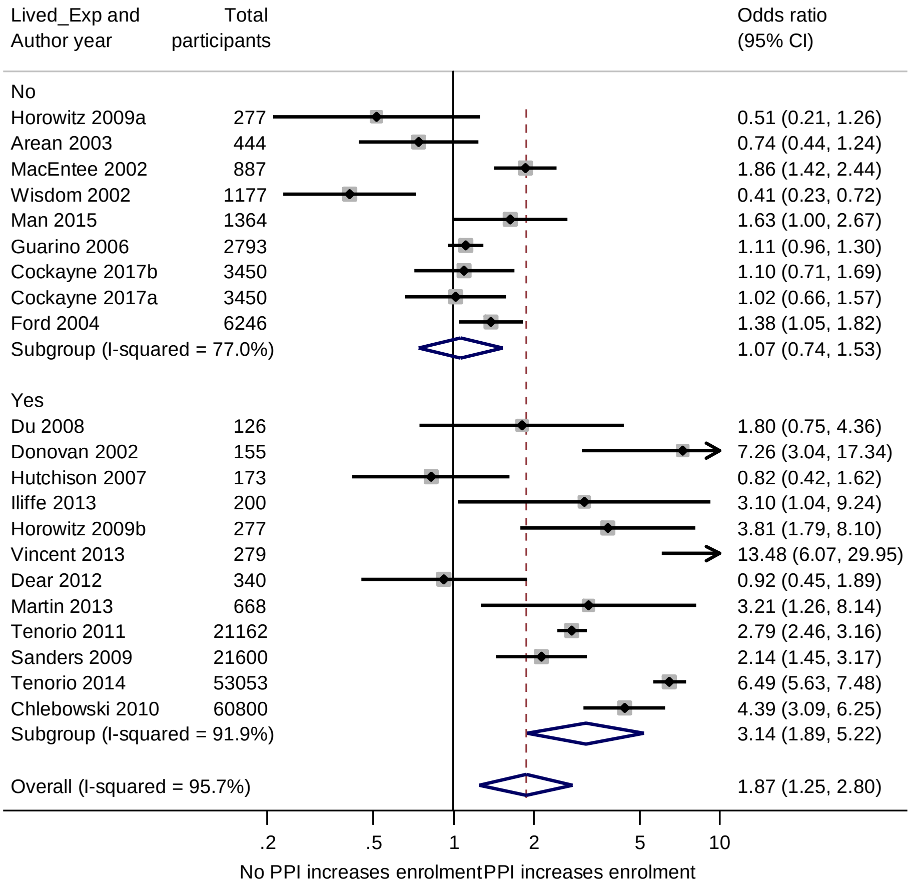

(k) PPI visible to potential trial participants

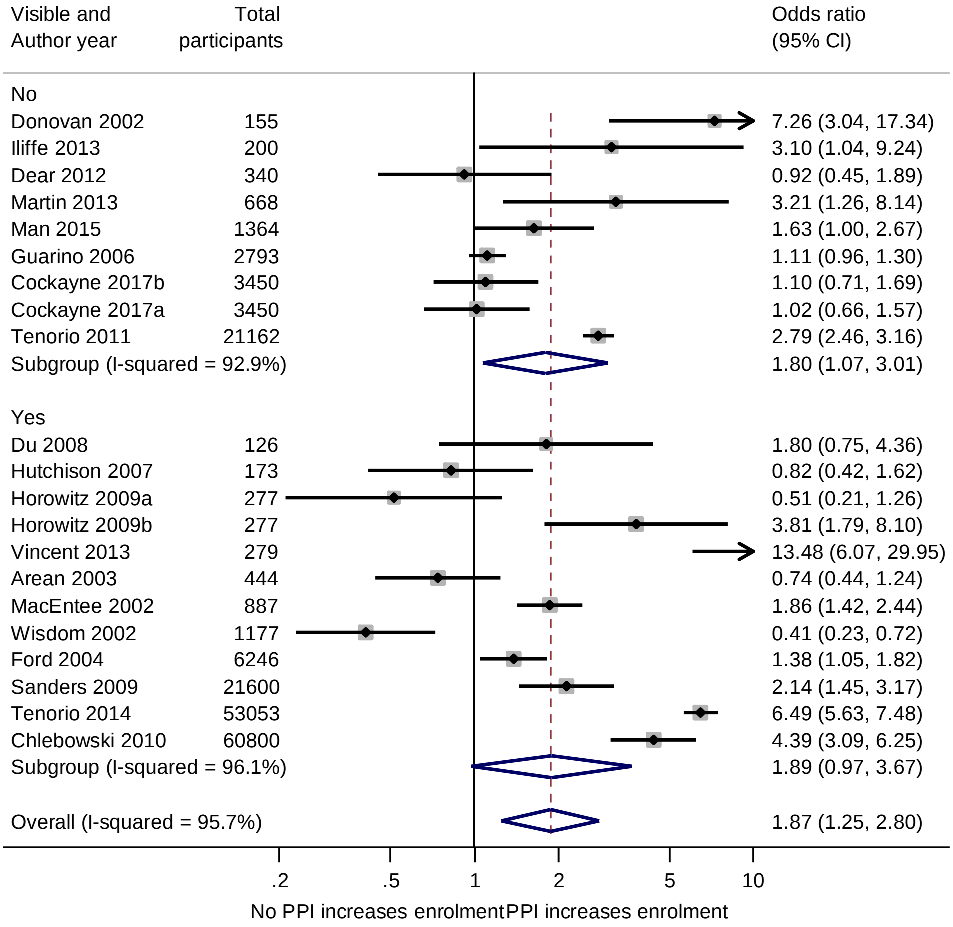

Appendix 3: Results of sensitivity analyses for enrolment outcome

a) Main analysis (randomised studies only)

Sensitivity analysis	Number of comparisons remaining (out of total 8)	Estimated OR (95% CI)	p-value
Excluding studies at high risk of bias	7	1.17 (1.01-1.35)	0.036
Excluding small studies (N<100)	8	1.16 (1.01-1.34)	0.035
Excluding PPI interventions with non-PPI components	1	-	-
Excluding formal qualitative research interventions	8	1.16 (1.01-1.34)	0.035
Excluding studies using a proxy denominator to measure enrolment rate	8	1.16 (1.01-1.34)	0.035

b) Secondary analysis (randomised and non-randomised studies combined)

Sensitivity analysis	Number of comparisons remaining (out of total 21)	Estimated OR (95% CI)	p-value
Excluding studies at high risk of bias	7	1.17 (1.01 – 1.32)	0.036
Excluding small studies (N<100)	21	1.87 (1.25 – 2.80)	0.004
Excluding PPI interventions with non-PPI components	6	2.70 (0.83 – 8.84)	0.084
Excluding formal qualitative research interventions	20	1.77 (1.18 – 2.64)	0.008
Excluding studies using a proxy denominator to measure enrolment rate	18	1.63 (1.06 – 2.52)	0.029

Appendix 4: Forest plot showing odds ratios for participant retention with vs. without PPI intervention (randomised studies only)

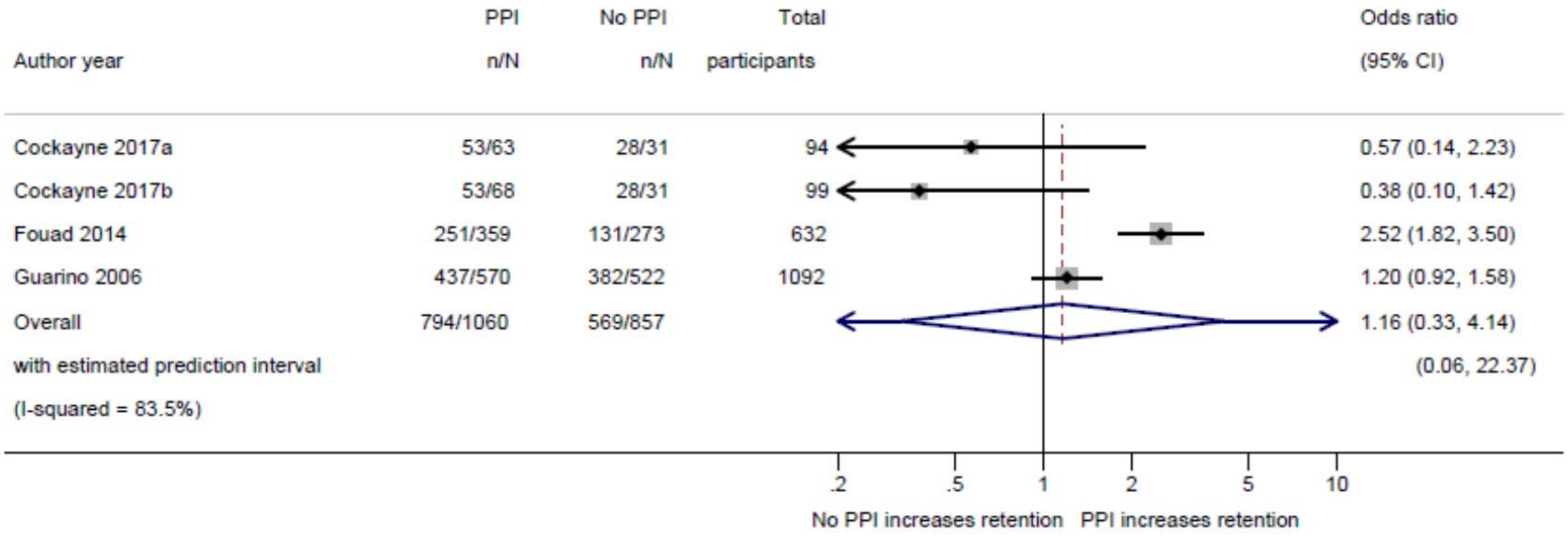

Appendix 5: Forest plot showing odds ratios for participant retention with PPI intervention vs. no PPI or non-PPI intervention (randomised and non-randomised studies combined)

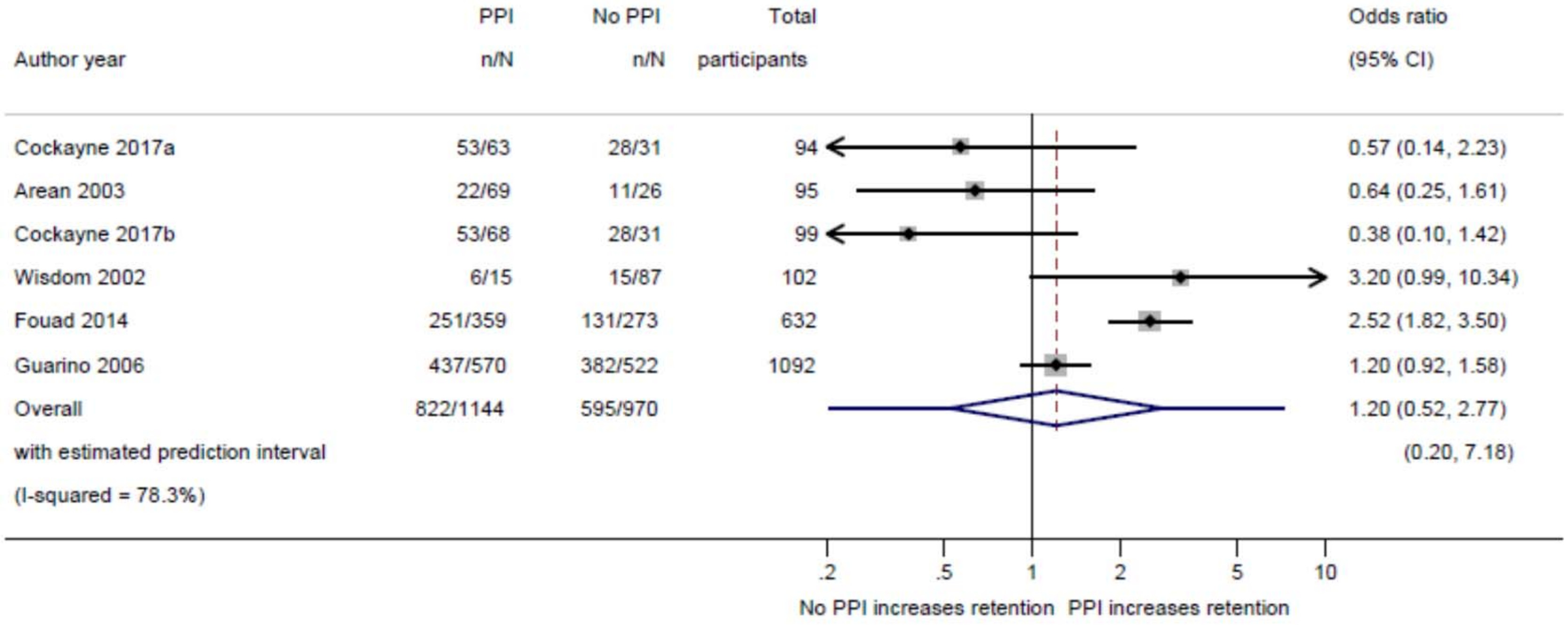

Appendix 6: Results of sensitivity analyses for retention outcome

a) Main analysis (randomised studies only)

Sensitivity analysis	Number of comparisons remaining (out of 4 total)	Estimated OR (95% CI)	p-value
Excluding studies at high risk of bias	4	1.16 (0.33 – 4.14)	0.727
Excluding small studies (N<100)	4	1.16 (0.33 – 4.14)	0.727
Excluding PPI interventions with non-PPI components	2	1.73 (0.02 – 188.33)	0.377
Excluding formal qualitative research interventions	4	1.16 (0.33 – 4.14)	0.727

b) Secondary analysis (randomised and non-randomised studies combined)

Sensitivity analysis	Number of comparisons remaining (out of 6 total)	Estimated OR (95% CI)	p-value
Excluding studies at high risk of bias	4	1.16 (0.33 – 4.14)	0.727
Excluding small studies (N<100)	5	1.36 (0.50 – 3.73)	0.445
Excluding PPI interventions with non-PPI components	2	1.73 (0.02 – 188.33)	0.377
Excluding formal qualitative research interventions	6	1.20 (0.52 – 2.77)	0.590
